# Supplementary material for: Polymorphic residues in HLA-B that mediate HIV control distinctly modulate peptide interactions with both TCR and KIR molecules
Source: Structure. 2024 Aug 8;32(8):1121–1136.e5. doi: 10.1016/j.str.2024.04.015 (PMC11329236; doi:10.1016/j.str.2024.04.015)
Supplement: Document S1. Figures S1–S7 and Tables S1 and S2 [file mmc1.pdf]

Structure, Volume *32*

Supplemental Information

Polymorphic residues in *HLA-B* that mediate HIV control distinctly modulate peptide interactions with both TCR and KIR molecules

Rhoda Tano-Menka, Nishant K. Singh, Itai Muzhingi, Xiaolong Li, Michael V. Mandanas, Clarety Kaseke, Charles R. Crain, Angela Zhang, Funsho J. Ogunshola, Liza Vecchiarello, Alicja Piechocka-Trocha, Arman Bashirova, Michael E. Birnbaum, Mary Carrington, Bruce D. Walker, and Gaurav D. Gaiha

HLA-B*5701 AA Mutation(s)	Notable HLA Alleles with Mutant Amino Acids
M67Y	B*0702
M67F	B*0801, B*3501
M67S	B*5201, Cw*07
M67A	Ala Mutant
S70Q	B*0702, B*0801
S70N	B*0801, B*1402, B*3501, Cw*0701
S70K	B*2705
S70A	Ala Mutant
V97S	B*0702, B*0801
V97R	B*3501, B*5801
V97T	B*5201
V97A	Ala Mutant
M67YS70Q	B*0702
M67YV97S	B*0702
S70QV97S	B*0702
M67YS70QV97S	B*0702
L156R	B*0702

Table S1. Mutations engineered in HLA-B*5701, related to Figures 2-8. The table depicts the engineered mutations in the HLA-B*5701 lentiviral expression plasmid and the protective and risk alleles in which these amino acid residues are naturally found. Each amino acid was also mutated to alanine.

AA Mutation	Forward Primer (Mutant)	Reverse Primer	Ta (◦C)
M67Y	GACACGGAACTATAAGGCCTCCGCGCAG	TCCCCGTCCCAATACTCC	63
M67F	GACACGGAACTTTAAGGCCTCCG	TCCCCGTCCCAATACTCC	65
M67S	GACACGGAACTCTAAGGCCTCCGC	TCCCCGTCCCAATACTCC	67
M67A	GACACGGAACGCAAAGGCCTCCGC	TCCCCGTCCCAATACTCC	63
S70Q	CATGAAGGCCCAGGCGCAGACTTACCG	TTCCGTGTCTCCCCGTCC	63
S70N	CATGAAGGCCAATGCGCAGACTTACCGAG	TTCCGTGTCTCCCCGTCC	71
S70K	CATGAAGGCCAAAGCGCAGACTTACCGAG	TTCCGTGTCTCCCCGTCC	67
S70A	CATGAAGGCCGCTGCGCAGACTTAC	TTCCGTGTCTCCCCGTCC	69
V97S	CATCATCCAGCGAATGTATGGCTGCGACGTGG	TGAGACCCGGCCTCGCTC	60
V97R	CATCATCCAGTCTATGTATGGCTGCGACGTGGG	TGAGACCCGGCCTCGCTC	60
V97T	CATCATCCAGACTATGTATGGCTGCGACGTGG	TGAGACCCGGCCTCGCTC	60
V97A	CATCATCCAGGCAATGTATGGCTGCGACG	TGAGACCCGGCCTCGCTC	60
M67YS70Q	GACACGGAACTATAAGGCCTCCGCGCAG	TCCCCGTCCCAATACTCC	63
M67YV97S	CATCATCCAGCGAATGTATGGCTGCGACGTGG	TTCCGTGTCTCCCCGTCC	60
S70QV97S	CATCATCCAGCGAATGTATGGCTGCGACGTGG	TTCCGTGTCTCCCCGTCC	60
M67YS70QV97S	CATCATCCAGCGAATGTATGGCTGCGACGTGG	TTCCGTGTCTCCCCGTCC	60

Table S2. Primers utilized to engineer mutations in HLA-B*5701, related to Figures 2-8. The table depicts the forward and reverse primers utilized to engineer mutations in the HLA-B*5701 lentiviral expression plasmid. Mutant codons highlighted in red text. Of note, the L156R mutation was engineered by synthesizing a mutant HLA-B*5701 DNA fragment and cloning into either the lentiviral expression vector or soluble HLA-B*5701 bacterial expression vector.

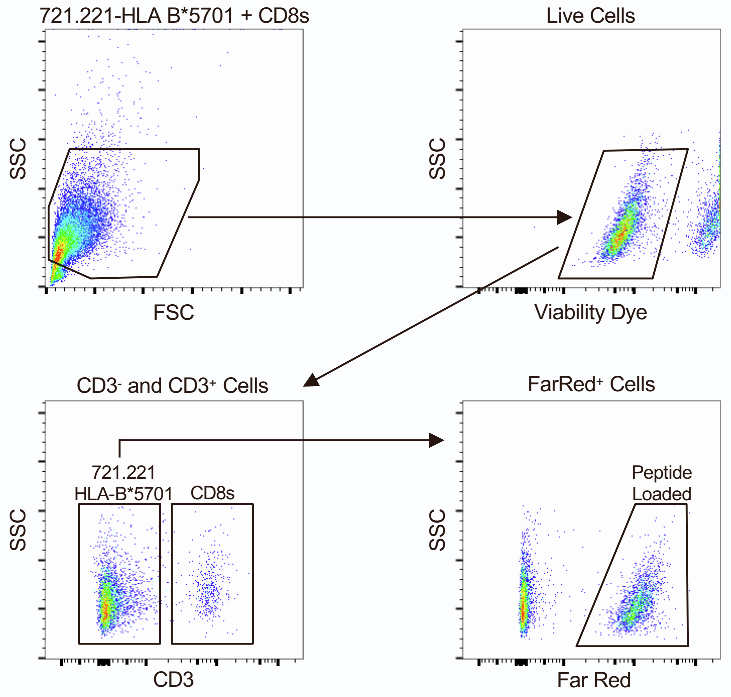

Figure S1. Flow cytometry gating strategy for CD8+ T cell elimination assay, related to Figures 3 and 8. HIV-specific CD8+ T cell clones that target an HLA-B*5701 epitope and 721.221-B*5701 wild-type and mutant cell lines were stained with viability dye and anti-CD3-PE antibody. 721.221-B*5701 cells were gated by absence of CD3 expression in the viable cell population. Peptide-loaded 721.221-B*5701 wild-type and mutant cells were subsequently gated by positive FarRed staining. The percentage decrease of the peptide-loaded FarRed population was used to determine the specific elimination of target 721-221-B*5701 cells by CD8+ T cell clones.

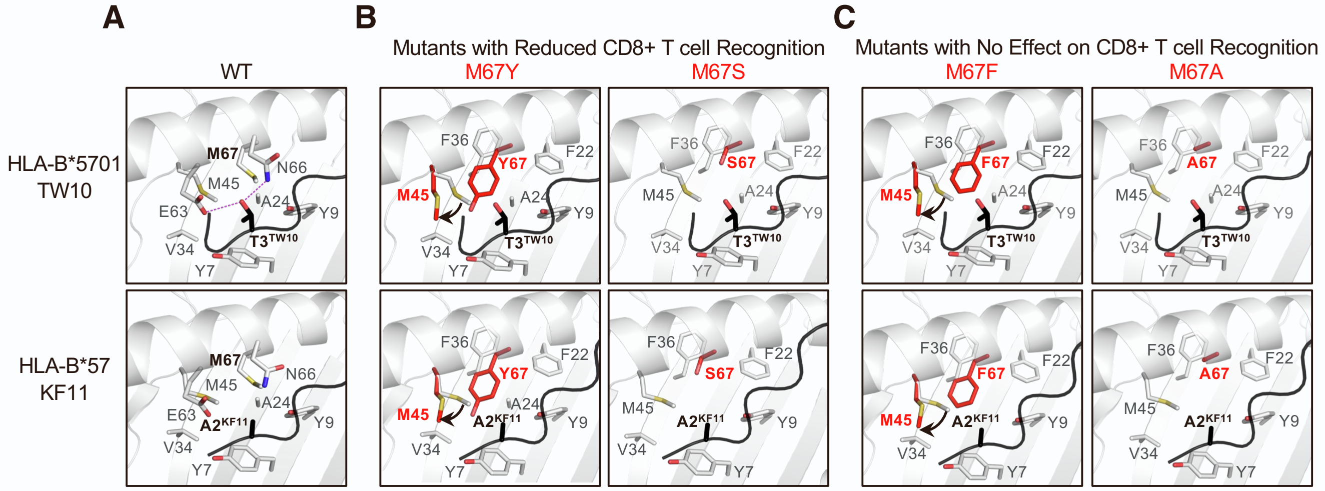

Figure S2. Structural analysis of HLA-B*57 wildtype and M67 mutants in complex with the TW10 and KF11 peptide, related to Figure 5. (A) Structural analysis of wild-type HLA-B*5701 presenting TW10 (PDB: 5V5M) and the closely related HLA-B*5703 presenting KF11 (PDB: 2YPK). HLA-B*57 was shown as grey ribbon and stick, while peptides were shown in black ribbon and stick. **(B)** Structural analysis of mutations that significantly affected CD8$^+$ T cell recognition and elimination (M67Y, M67S; **Figures 1-3**). The HLA-B*0702 structure (PDB: 5EO0) used to model Y67 and the Mutagenesis and Backbone Rotamers function in Pymol was used to model M67S. **(C)** Structural analysis of mutations that had no effect of CD8$^+$ T cell recognition and elimination (M67F, M67A; **Figures 1-3**). The HLA-B*3501 structure (PDB: 3LKO) was used to model M67F and the Mutagenesis and Backbone Rotamers function in Pymol was used to model M67A.

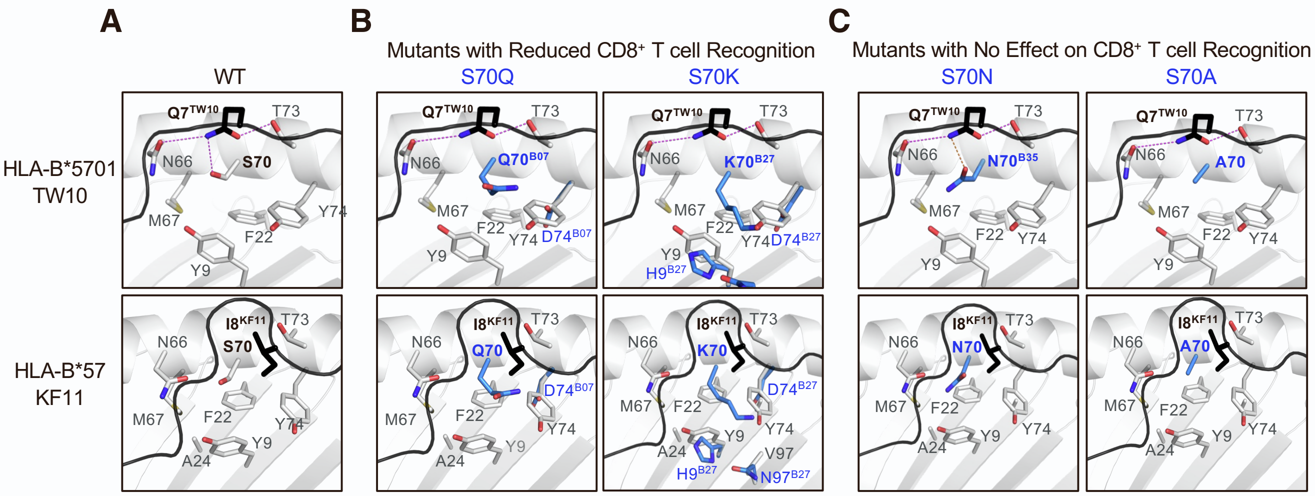

Figure S3. Structural analysis of HLA-B*57 wildtype and S70 mutants in complex with the TW10 and KF11 peptide, related to Figure 6. (A) Structural analysis of wild-type HLA-B*5701 presenting TW10 (PDB: 5V5M) and the closely related HLA-B*5703 presenting KF11 (PDB: 2YPK). HLA-B*57 was shown as grey ribbon and stick, while peptides were shown in black ribbon and stick. **(B)** Structural analysis of mutations that significantly affected $CD8^{+}$ T cell recognition and elimination (S70Q, S70K; **Figures 1-3**). The HLA-B*0702 structure (PDB: 5EO0) used to model Q70 and the HLA-B*2705 structure (PDB: 4G9D) was used to model K70. **(C)** Structural analysis of mutations that had no effect of $CD8^{+}$ T cell recognition and elimination (S70N, S70A; **Figures 1-3**). The HLA-B*3501 structure (PDB: 3LKO) was used to model S70N and the Mutagenesis and Backbone Rotamers function in Pymol was used to model S70A.

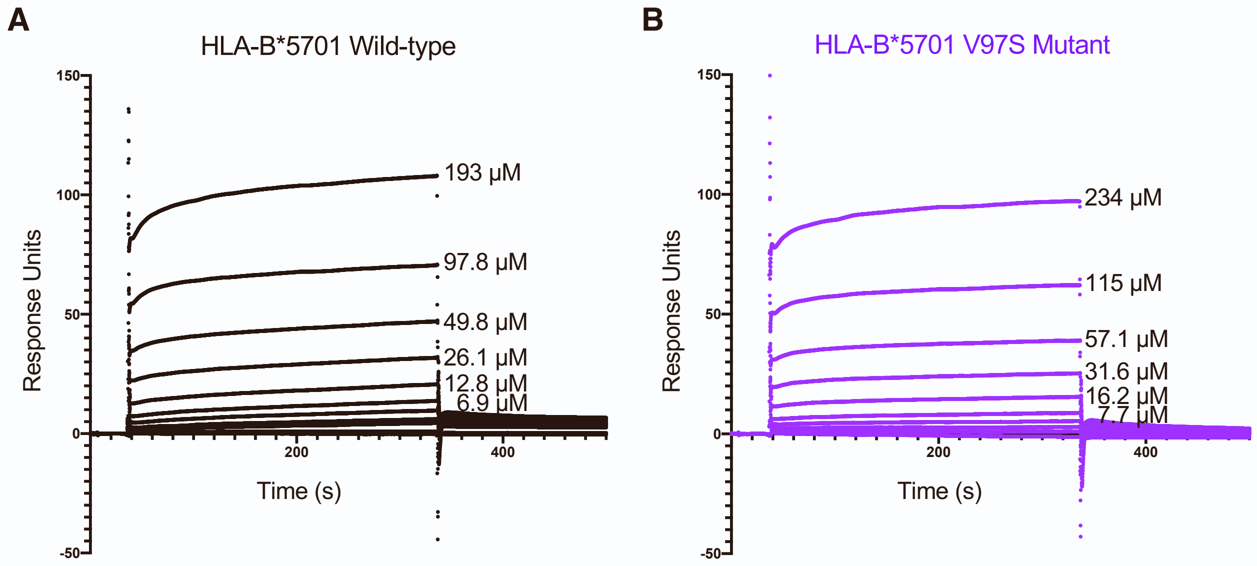

Figure S4. Representative surface plasmon resonance (SPR) of soluble HLA-B*5701 wild-type and mutant V97S monomers to immobilized KIR3DL1-Fc, related to Figure 7. (A) Representative surface plasmon resonance (SPR) injection series for soluble HLA-B*5701-TW10 complexes binding to immobilized KIR3DL1-Fc KIR3DL1 at range of concentrations, with a peak concentration of 193μM. **(B)** Representative surface plasmon resonance (SPR) injection series for soluble HLA-B*5701 V97S mutant-TW10 complexes binding to immobilized KIR3DL1-Fc at a range of concentrations, with a peak concentration of 234μM.

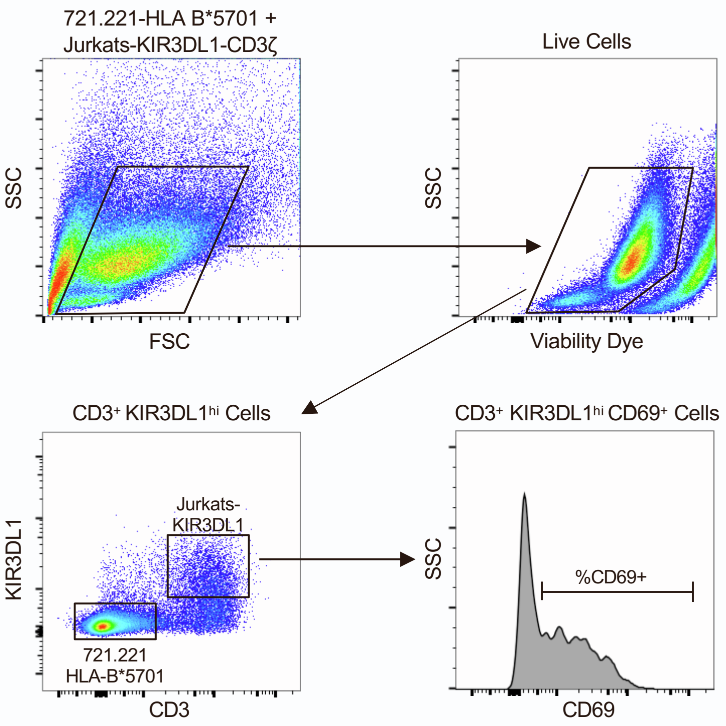

Figure S5. Flow cytometry gating strategy for Jurkat-KIR3DL1-CD3ζ reporter cell assay, related to Figures 7 and 8. Jurkat-KIR3DL1-CD3ζ cells and 721.221-B*5701 cell lines (wild-type and mutant) were stained with viability dye, anti-CD3-PE, anti-KIR3DL1-APC and anti-CD69-FITC antibodies. Jurkat-KIR3DL1-CD3ζ cells were identified by gating on KIR3DL1^{hi}CD3^{+} cells in the viable cell population. Binding to KIR3DL1 was determined by percentage of KIR3DL1^{hi}CD3^{+} that expressed CD69.

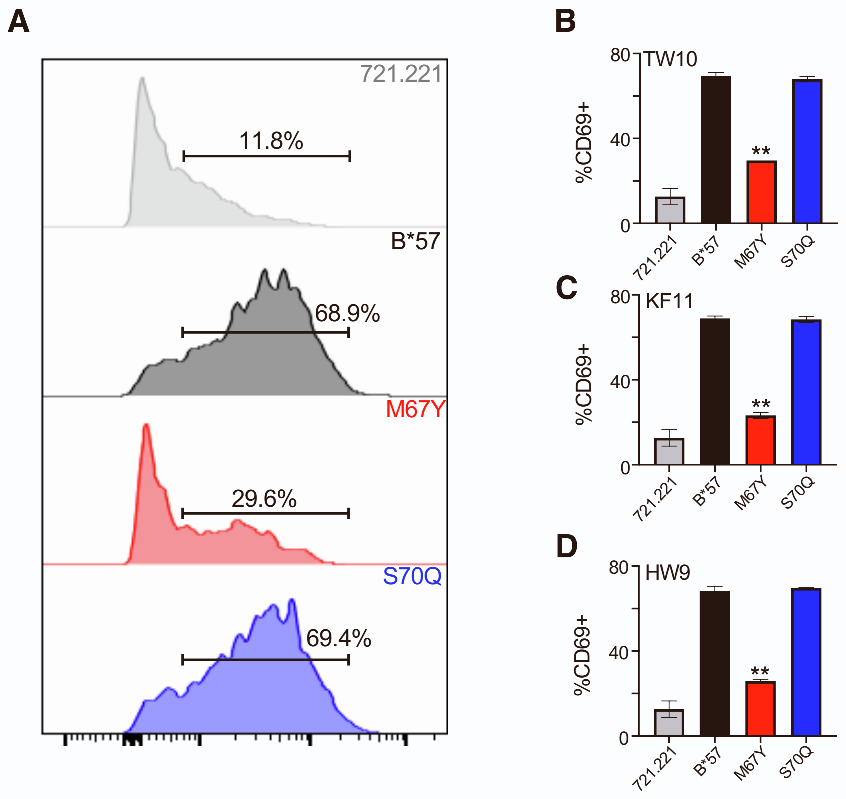

Figure S6. Effect of B*5701 M67 and S70 mutations on KIR3DL1 interactions, related to Figure 7. (A) Representative flow histograms of CD69 expression on CD3⁺ KIR3DL1⁺ Jurkat cells following co-culture with HLA-null 721.221 cells (gray), HLA-B*5701 cells (black), mutant M67Y (red) or mutant S70Q (blue) cells pulsed with TW10 peptide. (B-D) Comparison of %CD69⁺ CD3⁺ KIR3DL1⁺ Jurkat cells following co-culture with 721.221, wild-type HLA-B*5701, M67Y or S70Q mutant HLA-B*5701-expressing cell lines pulsed with TW10 peptide, KF11 peptide or HW9 peptide respectively. Error bars for (B-D) indicate standard deviation from three biological replicates performed independently. Statistical comparisons were made using an unpaired t-test.

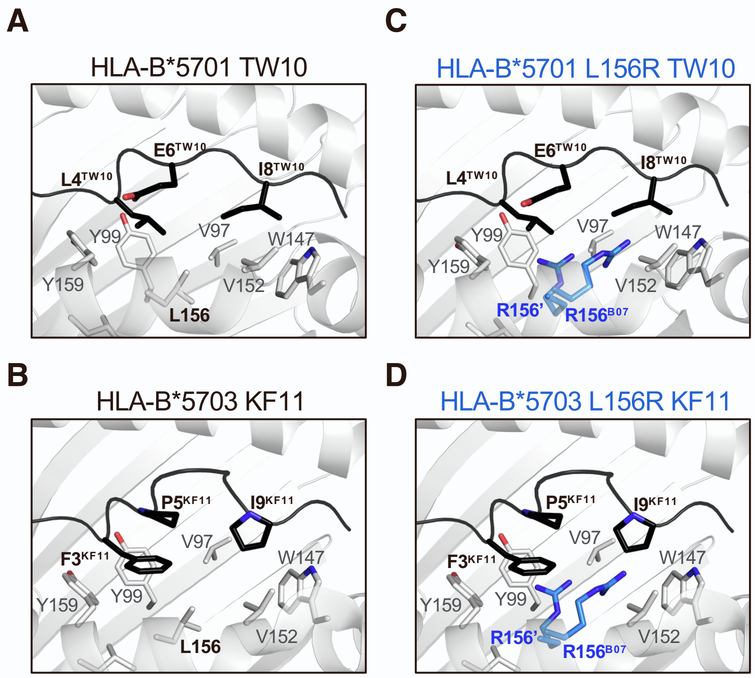

Figure S7. Structural analysis of HLA-B*57 wildtype and L156R mutants in complex with the TW10 and KF11 peptides, related to Figure 8. (A) Structural analysis of wild-type HLA-B*5701 presenting TW10 (PDB: 5V5M) and **(B)** the closely related wild-type HLA-B*5703 presenting KF11 (PDB: 2YPK). HLA-B*57 was shown as grey ribbon and stick, while peptides were shown in black ribbon and stick. **(C)** Structural analysis of mutant L156R HLA-B*5701-TW10 complex and **(D)** mutant L156R HLA-B*5703-KF11 complex. Two possible models of the L156R mutation were shown as blue sticks. The model of $R156^{B07}$ was constructed using the risk allele HLA-B07 (PDB: 5EO0), while the R156' model was constructed by using the Backbone Independent Rotamers function in Pymol.
