# Supplementary material for: Polymorphic residues in HLA-B that mediate HIV control distinctly modulate peptide interactions with both TCR and KIR molecules
Source: Structure. 2024 Aug 8;32(8):1121–1136.e5. doi: 10.1016/j.str.2024.04.015 (PMC11329236; doi:10.1016/j.str.2024.04.015)
Supplement: Document S2. Article plus supplemental information [file mmc2.pdf]

Structure

Article

# Polymorphic residues in *HLA-B* that mediate HIV control distinctly modulate peptide interactions with both TCR and KIR molecules

## Graphical abstract

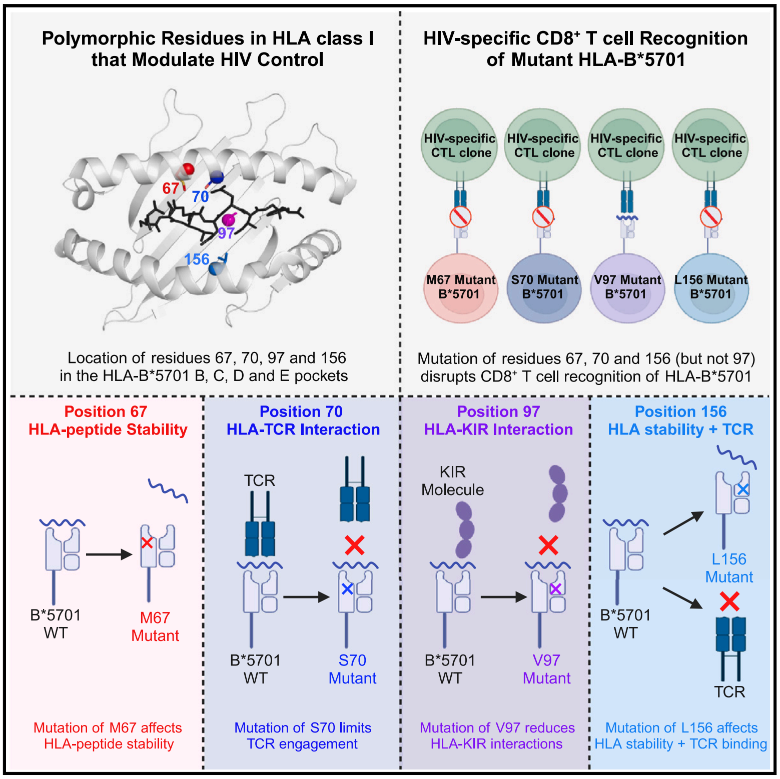


## Highlights

- Mutation of M67, S70, and L156, but not V97, in B*5701 affects CD8$^+$ T cell recognition
- Thermal denaturation analysis reveals that M67 and L156 maintain HLA-peptide stability
- Tetramer staining of HIV-specific CD8$^+$ T cells reveals role of S70 in HLA-TCR binding
- Mutation of V97 modulates interactions between HLA-B*5701 and inhibitory KIR3DL1

## Authors

Rhoda Tano-Menka, Nishant K. Singh, Itai Muzhingi, ..., Mary Carrington, Bruce D. Walker, Gaurav D. Gaiha

## Correspondence

ggaiha@mgh.harvard.edu

## In brief

Tano-Menka et al. characterized the functional roles of polymorphic residues in human leukocyte antigen (HLA) class I important for HIV outcomes. Evaluation of the protective allele HLA-B*5701 showed that these residues distinctly modulate HLA class I-peptide stability, T cell receptor recognition, interactions between HLA-peptide complexes, and killer inhibitor receptor molecules.

Tano-Menka et al., 2024, Structure *32*, 1121–1136
August 8, 2024 © 2024 The Author(s). Published by Elsevier Inc.
https://doi.org/10.1016/j.str.2024.04.015

CellPress

Structure

CellPress
OPEN ACCESS

Article

# Polymorphic residues in *HLA-B* that mediate HIV control distinctly modulate peptide interactions with both TCR and KIR molecules

Rhoda Tano-Menka,[1,10] Nishant K. Singh,[1,2,3,10] Itai Muzhingi,[1,10] Xiaolong Li,[1,4,10] Michael V. Mandanas,[2] Clarety Kaseke,[1] Charles R. Crain,[1] Angela Zhang,[1] Funsho J. Ogunshola,[1,3] Liza Vecchiarello,[1] Alicja Piechocka-Trocha,[1,3] Arman Bashirova,[5,6] Michael E. Birnbaum,[1,2,7] Mary Carrington,[1,5,6] Bruce D. Walker,[1,3,8] and Gaurav D. Gaiha[1,9,11,*]

[1]Ragon Institute of MGH, MIT and Harvard, Cambridge, MA 02139, USA
[2]Koch Institute for Integrative Cancer Research at MIT, Cambridge, MA 02142, USA
[3]Howard Hughes Medical Institute, Chevy Chase, MD 20815, USA
[4]The First Affiliated School of Life Sciences, Division of Life Sciences and Medicine, University of Science and Technology of China, Hefei, Anhui 230027, China
[5]Basic Science Program, Frederick National Laboratory for Cancer Research, National Cancer Institute, Frederick, MD 21702, USA
[6]Laboratory of Integrative Cancer Immunology, Center for Cancer Research, National Cancer Institute, Bethesda, MD 20892, USA
[7]Department of Biological Engineering, Massachusetts Institute of Technology, Cambridge, MA 02139, USA
[8]Institute for Medical Engineering and Science and Department of Biology, Massachusetts Institute of Technology, Cambridge, MA 02139, USA
[9]Division of Gastroenterology, Massachusetts General Hospital, Boston, MA 02114, USA
[10]These authors contributed equally
[11]Lead contact
*Correspondence: ggaiha@mgh.harvard.edu
https://doi.org/10.1016/j.str.2024.04.015

## SUMMARY

Immunogenetic studies have shown that specific HLA-B residues (67, 70, 97, and 156) mediate the impact of HLA class I on HIV infection, but the molecular basis is not well understood. Here we evaluate the function of these residues within the protective *HLA-B*5701* allele. While mutation of Met67, Ser70, and Leu156 disrupt CD8$^+$ T cell recognition, substitution of Val97 had no significant impact. Thermal denaturation of HLA-B*5701-peptide complexes revealed that Met67 and Leu156 maintain HLA-peptide stability, while Ser70 and Leu156 facilitate T cell receptor (TCR) interactions. Analyses of existing structures and structural models suggested that Val97 mediates HLA-peptide binding to inhibitory KIR3DL1 molecules, which was confirmed by experimental assays. These data thereby demonstrate that the genetic basis by which host immunity impacts HIV outcomes occurs by modulating HLA-B-peptide stability and conformation for interaction with TCR and killer immunoglobulin receptor (KIR) molecules. Moreover, they indicate a key role for epitope specificity and HLA-KIR interactions to HIV control.

## INTRODUCTION

In the vast majority of individuals, untreated HIV infection leads to sustained viremia, CD4$^+$ T cell decline, and progression toward AIDS. However, in a small subset of individuals known as HIV controllers, plasma viremia is suppressed to below the transmission and progression threshold of 2,000 RNA copies/mL,[1–3] and in many instances to undetectable levels (“elite controllers”), making them compelling natural cases of functional HIV cure.[4] Studies of HIV cohorts have revealed that specific human leukocyte antigen-B (*HLA-B*) alleles (i.e., *HLA-B*5701* and *B*2705*)[5,6] are consistently enriched in HIV controllers, whereas other *HLA-B* alleles are strongly associated with rapid progression (*HLA-B*0702* and *B*3501*).[7–9] This strong influence of *HLA-B*[10] has also been the primary finding of several genome-wide association studies (GWAS) of HIV$^+$ individuals,[11–16] for which several competing mechanistic explanations have been proposed regarding the effect of HLA class I alleles on outcomes of HIV infection. These include variation in functional CD8$^+$ T cell targeting of specific conserved and constrained epitopes,[17–21] the fraction of the naive T cell repertoire specific for HIV,[22] CD8$^+$ T cell cross-reactivity,[23,24] functional avidity and antiviral activity of T cell receptors (TCRs),[25–27] and binding to leukocyte immunoglobulin like receptor (LILR)B2[28] and killer immunoglobulin receptors (KIRs).[29,30]

Determining mechanisms of HIV control among this list of possibilities has been a challenging venture. However, importantly, what the aforementioned immunogenetic studies revealed is that the effect of HLA-B on HIV outcomes can be fully attributed to a limited and distinct set of four polymorphic residues in the B,


This is an open access article under the CC BY license (http://creativecommons.org/licenses/by/4.0/).

C, D, and E-pockets,[12,15,16] providing a clear reductionist opportunity to determine mechanistic underpinnings. In an early GWAS study, residues at positions 67, 70, and 97 of HLA-B were found to be more strongly associated with HIV control and risk than any individual allele,[12] including HLA-B*5701, the strongest HLA determinant of viral load.[11] For example, certain HLA-B residues at positions 67, 70, and 97 (i.e., methionine 67, serine 70, and valine 97) were associated with the low viral loads, while residues (tyrosine 67, glutamine 70, and serine 97) were associated with high viral loads.[12] Interestingly, position 97 was found to have the most significant impact on HIV outcomes, with valine 97 having the strongest individual association with low setpoint viral load and viral control.[12,16,31] In a subsequent GWAS,[15] which analyzed a larger and more diverse multi-ancestry population, a novel association was made between HIV outcomes and polymorphic residue 156 in the D-pocket of HLA-B, in addition to re-establishing the importance of residues at positions 67 and 97. This collectively indicated that polymorphic residues 67, 70, 97, and 156 govern the molecular and genetic basis by which HLA-B modulates control of HIV infection, but the functional contributions of these residues remain poorly defined. Delineating the mechanisms that underlie these causal inferences from GWAS would therefore critically inform mechanisms of HIV control and provide key guidance for functional cure development.

Thus, in the study, we assessed the impact of systematically mutating these polymorphic residue positions 67 (Met; M), 70 (Ser; S), 97 (Val; V), and 156 (Leu; L) within the protective *HLA-B*5701* allele. Using wild type and mutant B*5701$^+$ cells as targets for HIV-specific CD8$^+$ T cells, we found that mutation of M67, S70, and L156 significantly affects CD8$^+$ T cell recognition of B*5701-restricted HIV epitopes, but surprisingly, there was no substantial impact following mutation of V97. Biochemical assays demonstrated that M67 and L156 affect HLA-B*5701-HIV peptide stability, while S70 and L156 disrupt binding of HLA-B*5701-peptide complexes with T cell receptors (TCRs). Structural analyses of the HLA-B*5701-TW10 epitope (Gag p24$_{108-117}$) complex suggested that V97 may impact HLA-B*5701-TW10 interactions with KIR3DL1, a key molecule of both the innate and adaptive response to HIV,[30,32,33] which we confirmed by protein-protein binding assessments and KIR3DL1 reporter cell assay.[34] Thus, among the numerous posited mechanisms, these data demonstrate that the primary manner by which the host immune response affects outcomes to HIV infection is by modulating the stability and confirmation of HLA-B-restricted viral peptides for recognition by HIV-specific TCR and KIR molecules. Given that a key differentiating factor of protective and risk alleles is the distinct set of epitopes that each HLA class I allele presents, these data further support the impact of CD8$^+$ T cell epitope specificity to HIV outcomes. Moreover, they reveal that the residue most significantly associated with HIV control (i.e., position 97) impacts interactions between HLA-bound peptides and KIR molecules.

## RESULTS

### Structural analysis of polymorphic HLA-B residue positions 67, 70, and 97 in HLA-B-5701-TW10 peptide complex

We first assessed the structural attributes of polymorphic residues M67, S70, and V97 in HLA-B*5701 given that the more recent findings on the importance of polymorphic 156 residue position had not yet been published[15] when we initiated this study. From a structural perspective, the variable residues of highly polymorphic HLAs predominantly occur in the peptide-binding groove, while residues present on the two alpha helices typically limit differential TCR docking patterns on the HLA-peptide surface. The residues situated within the peptide-binding groove dictate the formation of anchoring pockets A–F, crucial for accommodating diverse peptide repertoires. Within the context of HIV control and progression, the key polymorphic HLA class I residues at positions 67, 70, and 97[12] are clearly found in the peptide-binding groove in the previously solved structure of highly protective HLA-B*5701 allele in complex with the HIV Gag p24$_{108-117}$ TW10 (TSTLQEQIGW) peptide[35] (Figures 1A and 1B). At these critical positions, residues 67, 70, and 97 are vital components of pocket B, pocket C, and pocket E, respectively (Figure 1B). Using the structural confluence of the TW10-HLA-B*5701 complex as a reference, the TW10 residue T3 anchors its side chain into pocket B, Q7 invades pocket C with its side chain, while L8 introduces its side chain into pocket E. Thus, it can be inferred that the polymorphic HLA residues residing within these pockets are potentially vital determinants of peptide presentation. However, further investigation is required to discern the mechanistic basis by which these residues confer the protective effect to the HLA-B*57 allele.

### Effect of residue mutations at polymorphic HLA-B residue positions 67, 70, and 97 on HIV-specific CD8$^+$ T cell recognition and elimination

To assess the impact of mutations to residues M67, S70, and V97 in the HLA-B*5701 allele, we utilized a lentiviral plasmid in which the HLA-B*5701 heavy chains were linked to a ZsGreen reporter via an N-terminal self-cleaving P2A peptide and a C-terminal puromycin resistance gene via an internal ribosome entry sequence (IRES) (Figure 2A). We generated 12 single mutants (4 mutations per each amino acid position), three double mutants, and a single triple mutant, all of which were engineered by site-directed mutagenesis (Tables S1 and S2). The amino acids that we selected as substitutions for M67, S70, and V97 were all HLA allelic variants associated with significant relative increases in quantitative viral load in comparison to HLA-B*5701[12] or an alanine due to the inert and non-bulky nature of its side chain. The double mutants (M67YS70Q, M67YV97S, and S70QV97S) and triple mutant (M67YS70QV97S) all incorporated amino acid substitutions that were present in the risk allele *HLA-B*07:02* and were each individually associated with the highest relative quantitative viral loads in comparison to M67, S70, and V97.[12]

The constructs were transduced individually into the HLA-null human B cell line 721.221 and selected in the presence of puromycin. Surface expression of wild-type and mutant HLA-B*5701 molecules was detected and quantified by staining with the pan-HLA antibody W6/32,[36] and ZsGreen fluorescence was utilized as an internal measure of transgene expression (Figure 2B). The ratio of surface HLA to ZsGreen fluorescence was then calculated for each mutant HLA-B*5701 cell line and normalized to the B*5701 wild type. This revealed that two of the M67 single mutants (M67Y and M67S), both M67Y-containing double

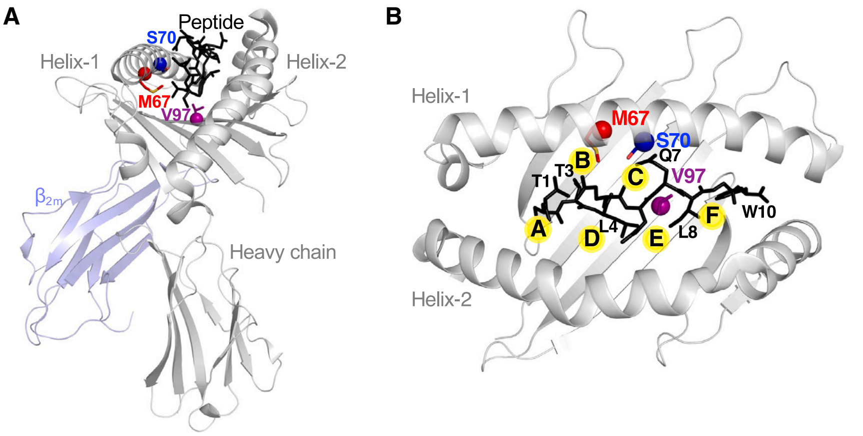


**Figure 1. Polymorphic residues 67, 70, and 97 are located in distinct pockets of the HLA class I peptide-binding groove**

(A) The overall view of the previously solved three-dimensional structure (PDB: 5V5M) of HLA-B*5701 bound to the TW10 (Gag p24$_{108-117}$) peptide (PDB: 5V5M). The gray ribbon represents the HLA-B*5701 heavy chain and the light blue ribbon represents $\beta_2$ microglobulin ($\beta_2$m). The black stick represents the TW10 peptide. The highlighted amino acid positions 67 (red), 70 (blue), and 97 (purple), which line the peptide-binding groove, are shown as spheres with residue side chains.

(B) The top-down view of the HLA-B*5701-TW10 complex. The HLA class I A-F pockets are circled in yellow.

mutants and the M67YS70QV97S triple mutant had significantly reduced relative surface HLA expression to ZsGreen (Figure 2C), suggesting a potential role for residue 67 in mediating HLA class I surface stability.

We next assessed the effect of mutations to M67, S70, and V97 in HLA-B*5701 on HIV-specific CD8$^+$ T cell recognition. We incubated wild-type and mutant cell lines with immunodominant B*5701-restricted HIV epitopes TW10 Gag p24$_{108-117}$, KAFSPEVIPMF (KF11) Gag p24$_{30-40}$ and HTQGYFPDW (HW9) Nef$_{116-124}$ that have previously been associated with HIV control[20,37] and assessed reactivity by cognate, patient-derived HIV-specific CD8$^+$ T cell clones using an IFN-$\gamma$ enzyme-linked immunospot (ELISpot) assay. This revealed that specific mutations at positions 67 (M67Y and M67S) and 70 (S70Q and S70K) in HLA-B*5701 but not any of the mutations at position 97, led to a significant reduction in HIV-specific CD8$^+$ T cell reactivity to TW10 (Figures 2D and 2E), KF11 (Figure 2F), and HW9 (Figure 2G). Interestingly, substitution of M67 and S70 with allelic polymorphisms associated with the highest relative quantitative viral load (M67Y and S70Q),[12] which are also present in the risk allele *HLA-B*0702*, had the greatest effect on HIV-specific CD8$^+$ T cell clone recognition, whether present as a single, double, or triple mutant (Figures 2D–2G).

In addition to CD8$^+$ T cell recognition, we also assessed the impact of mutations to residues 67, 70, and 97 in HLA-B*5701 on HIV-specific CD8$^+$ T cell cytolysis using a previously described flow cytometry-based CD8$^+$ T cell elimination assay.[38] HLA-B*5701 wild type and mutant cell lines were pulsed with TW10, KF11, or HW9 peptides prior to fluorescent labeling and mixing in a 1:1 ratio with non-fluorescent, non-peptide pulsed cells. These cell mixtures were then incubated for 16 h with a cognate HIV-specific CD8$^+$ T cell clone and the degree of epitope-specific target cell elimination was determined by the reduction in the frequency of fluorescently labeled peptide-pulsed cells (Figure S1). Application of this assay revealed that mutations to M67 (M67Y and M67S) and S70 (S70Q and S70K), but not V97, led to a significant reduction in target cell elimination by HIV-specific CD8$^+$ T cells for all three B*5701 epitopes (Figures 3A–3D), thereby providing additional confirmation of our CD8$^+$ T cell recognition assay findings. Moreover, we observed that the M67Y and S70Q mutations, which were associated with the highest quantitative viral load increase, had the greatest effect on CD8$^+$ T cell-mediated elimination of HLA-B*5701 target cells.

Assays that utilize HIV-specific CD8$^+$ T cell clones facilitate highly robust and reproducible assessments of target cell recognition and elimination but only in the context of a single T cell receptor (TCR). We therefore obtained primary CD8$^+$ T cells from 10 HIV-infected HLA-B*5701$^+$ controllers with detectable responses to the TW10 (4 responses), KF11 (4 responses), and HW9 (2 responses) epitopes to assess the impact of M67, S70, and V97 mutations on polyclonal CD8$^+$ T cell recognition. Given the effects of the M67Y and S70Q mutations on CD8$^+$ T cell clone recognition and elimination, we specifically focused on these substitutions, in addition to V97S (which is also present in the risk allele *B*0702*), when assessing polyclonal T cell recognition. This revealed that similarly to studies with CD8$^+$ T cell clones, mutations at positions 67 and 70, but not position 97, affect polyclonal CD8$^+$ T cell recognition when we performed a cumulative assessment of all three HIV peptide-loaded HLA-B*5701 expressing cells which facilitated robust statistical comparisons (Figures 4A and 4B). Collectively, these results indicate that some, but not all, HLA-B residues identified by immunogenetic studies impact CD8$^+$ T cell recognition and elimination.

## Effect of residue mutations at polymorphic HLA-B positions 67, 70, and 97 on HLA class I-peptide stability

Given that certain M67 and S70 mutations in HLA-B*5701 affect CD8$^+$ T cell recognition of HIV epitopes, and mutation of M67 also appears to decrease HLA class I surface expression (Figure 2C), we chose to investigate the impact of mutations at positions 67, 70, and 97 on HLA-B*5701-peptide stability as a putative biochemical mechanism for their role in modulating HIV control. This was accomplished by assessing thermal denaturation of soluble HLA class I-peptide complexes.[39] In this assay, soluble HLA class I monomers with bound peptide are incubated in the presence of an environmentally sensitive fluorescent molecule whose fluorescence is enhanced when bound to exposed hydrophobic surfaces during protein unfolding. As a result, the thermal stability ($T_m$, defined as the temperature at which 50% of the protein is unfolded) of an HLA class I-peptide complex

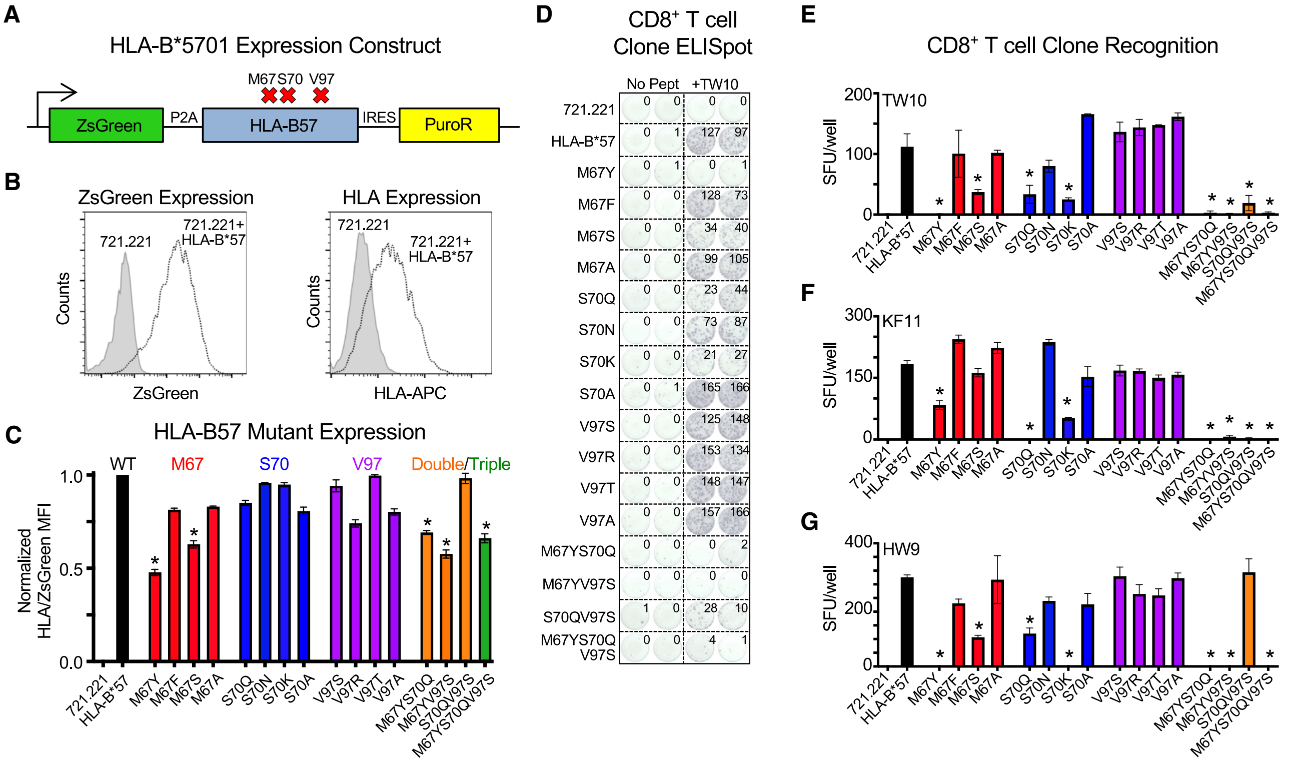


**Figure 2. Mutation of M67 and S70, but not V97, in HLA-B*5701 affects target cell recognition by HIV-specific CD8+ T clones**

(A) Schematic of HLA-B*5701 lentiviral construct utilized to generate wild-type and mutant B*5701 expressing 721.221 cell lines.

(B) Representative ZsGreen and HLA-B*5701 expression in non-transduced (721.221, filled gray) and transduced (721.221 + HLA-B*57, open dashed) cells.

(C) Ratio of surface HLA-B*5701 expression to ZsGreen transgene expression for B*5701 M67, S70, and V97 single, double and triple mutants normalized to wild-type HLA-B*5701-expressing cell line (black).

(D) Representative IFN-$\gamma$ ELISpot data of TW10-specific CD8+ T cell clone recognition of HLA-null 721.221 cells, wild-type B*5701-expressing 721.221 cells and mutant M67, S70, and V97 B*5701-expressing 721.221 cells incubated in the presence and absence of TW10 peptide.

(E) Comparison of IFN-$\gamma$ ELISpot spot forming units (SFU) per well following recognition of peptide-pulsed 721.221, wild-type and mutant HLA-B*5701-expressing cell lines by TW10-specific CD8+ T cell clone, (F) KF11-specific CD8+ T cell clone and (G) HW9-specific CD8+ T cell clone. Single mutations of M67, S70, and V97 are arranged in order from left to the right in terms of their association with higher to lower viral loads by GWAS.[12] Statistical comparisons were made using an unpaired t test to the 721.221 HLA-B*57 wild-type-expressing control cells. Error bars for (C), (E), (F), and (G) indicate standard deviation from three biological replicates performed independently. Calculated $p$-values were as follows: *$p < 0.05$.

can be determined by fluorometric measurements during incremental temperature increases, which serve as a proxy for the stabilizing capacity of the bound epitope.[40]

We therefore expressed and refolded soluble monomers for HLA-B*5701 wild type, the M67Y, S70Q, and V97S single mutants and the M67YS70Q, M67YV97S, and S70QV97S double mutants in complex with the TW10, KF11, or HW9 peptides. Thermal denaturation of these HLA-peptide complexes revealed that mutation of M67, whether as a single or double mutant, significantly affects HLA class I-peptide stability for all three peptides, as demonstrated by decreases in $T_m$ of 19°C for TW10, 18°C for KF11, and 16°C for HW9 for single M67Y mutants (Figure 5A) and comparable decreases for double mutants harboring an M67Y mutation (Figures 5B–5D). In contrast, mutation of S70 or V97 as either a single or double mutant (S70QV97S) led to only modest, non-significant decreases in $T_m$ for all three epitopes (Figures 5A–5D).

Structural analysis of the previously solved crystal structure of HLA-B*5701 bound by TW10[35] (PDB: 5V5M) reveals that M67 does not directly engage with the HIV peptide, but appears to maintain the appropriate hydrophobic microenvironment, along with Ala24, Val34, Met45 and the aromatic rings of Tyr7 and Tyr9. This facilitates the interaction between the HLA molecule and the Thr3 anchor residue of TW10, with the hydrophobic portion of Thr3 remaining buried in the hydrophobic microenvironment, and the hydrophilic portion forming two hydrogen bonds with Glu63 and Met66 (Figure 5C). Interestingly, residues at position 45, 63, and 66 in HLA molecules have also been previously identified in immunogenetics studies to be associated with viral control,[12,13] although not as strongly as those at position 67. *In silico* mutation of M67 to Tyr (Y) using a previously solved HLA-B*0702 structure as template (PDB: 5EO0, which naturally has a Tyr at position 67) reveals that this mutation leads to substantial disruption of the hydrophobic B-pocket microenvironment due to the hydrophilic hydroxyl group on the Tyr side chain (Figure 5D), which likely explains its effect on HLA-peptide stability and subsequent T cell recognition. This is supported by the observation that substitution of M67 to Phe (F) leads to similar changes in neighboring side chains in comparison to M67Y (notably for M45) for both HLA-B*5701-TW10 and the closely related

CellPress
OPEN ACCESS

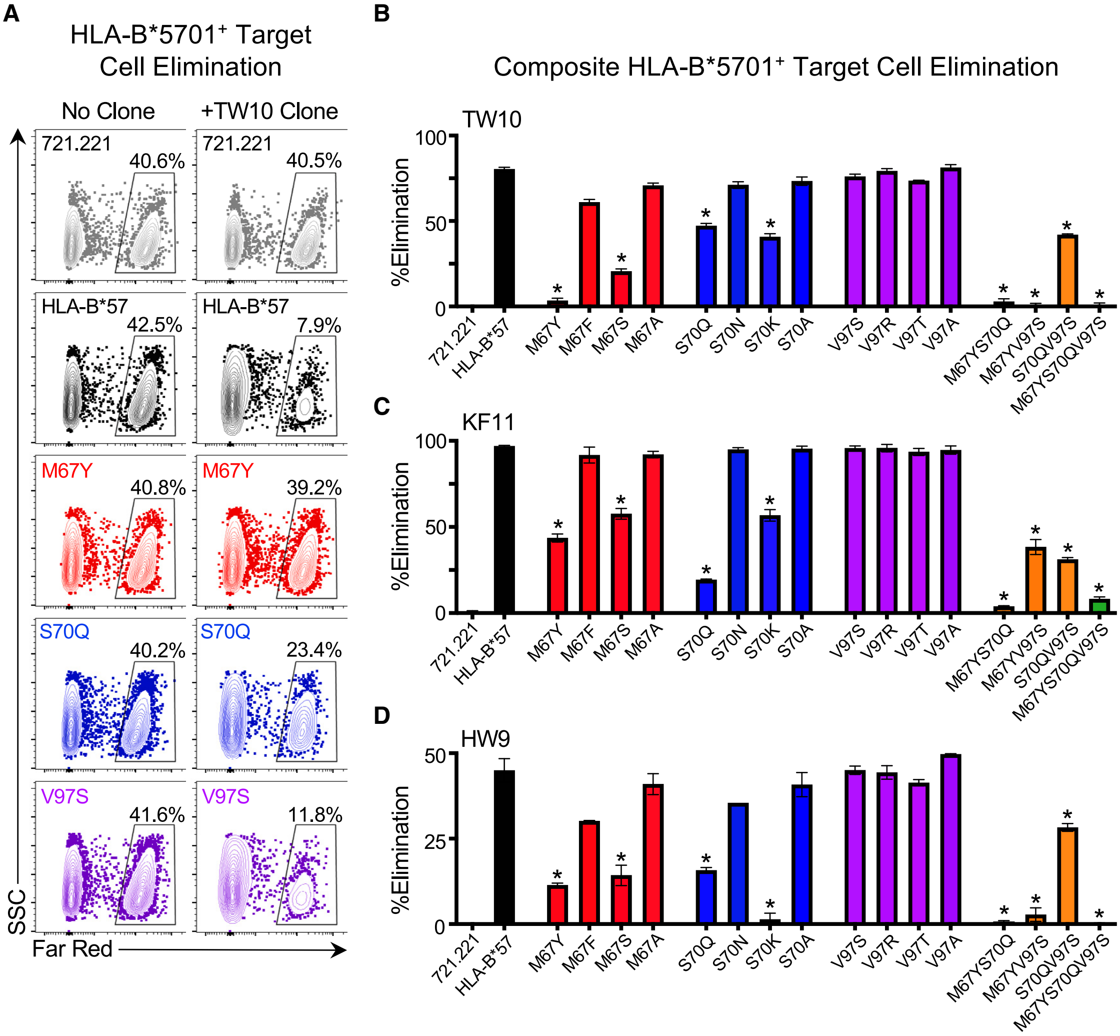


**Figure 3. Mutation of M67 and S70, but not V97, affects elimination of HLA-B*5701 target cells by HIV-specific CD8+ T clones**

(A) Representative CD8+ T cell elimination of peptide-pulsed HLA-null 721.221, HLA-B*5701 wild-type, M67Y, S70Q and V97S single mutants following co-culture with and without a TW10-specific CD8+ T cell clone. 50% of target cells were loaded with TW10 peptide and stained with CellTrace Far Red and subsequently mixed 1:1 with unpulsed, non-stained target cells, prior to overnight co-culture with TW10-specific CD8+ T cell clone. %Elimination was determined by the following formula: (1 - (%FarRed+ with CD8+ T cell clone/%FarRed+ without CD8+ T cell clone).

(B) Comparison of %elimination of peptide-loaded 721.221, wild-type and mutant HLA-B*5701-expressing cell lines by TW10-specific CD8+ T cell clone, (C) KF11-specific CD8+ T cell clone and (D) HW9-specific CD8+ T cell clone. Statistical comparisons were made using an unpaired t test to the 721.221 HLA-B*57 wild-type-expressing control cells. Error bars for (B–D) indicate standard deviation from three biological replicates performed independently. Calculated *p*-values were as follows: *$p < 0.05$.

HLA-B*5703-KF11[41] peptide complex (Figure S2) but has no significant effect on CD8+ T cell recognition and elimination (Figures 2 and 3). Moreover, we observed a similar difference between the M67S and M67A substitutions, with the hydrophilic Ser mutation having a significant effect on surface HLA expression (Figure 2C) and CD8+ T cell recognition (Figure 2) that was not observed for the hydrophobic Ala mutation, despite comparable occupancy of the residue sidechains (Figure S2). Collectively, these data suggest the polymorphic residue 67 position in HLA-B modulates outcomes to HIV infection through its role within the HLA B-pocket microenvironments and the downstream effects on the stability of HLA class I-viral peptide complexes.

## Effect of residue mutation at polymorphic HLA-B position 70 on TCR binding to HLA class I-peptide complexes

Given that mutation of S70 had only a minimal effect on HLA class I-peptide stability (Figure 5) but a substantial effect on CD8+ T cell recognition and elimination (Figures 2, 3, and 4), we evaluated whether substitution of S70 affected the biochemical interaction between HLA class I-peptide complexes and TCRs. We therefore generated fluorescently labeled wild-type and mutant HLA B*5701-peptide tetramers, with specific focus on the damaging S70Q mutation and the immunodominant TW10 epitope, and assessed their ability to bind to three distinct, patient-derived TW10-specific CD8+ T cell clones. This revealed

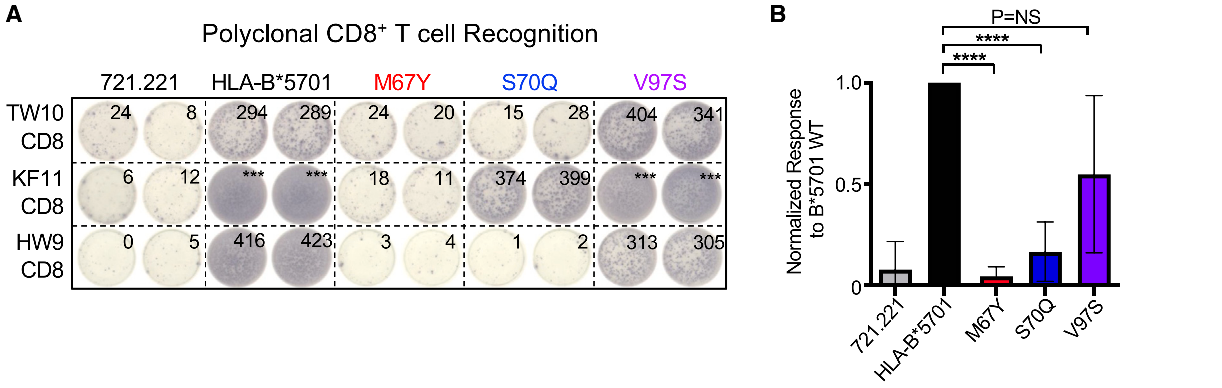


**Figure 4. Mutation of residues M67 and S70, but not V97, affects target cell recognition by *ex vivo* polyclonal HIV-specific CD8+ T cells**
(A) Representative IFN-γ ELISpot data of polyclonal TW10-, KF11-, and HW9-specific CD8+ T cell recognition of HLA-null 721.221 cells, wild-type B*5701-expressing 721.221 cells, and mutant M67, S70, and V97 B*5701-expressing 721.221 cells incubated in the presence and absence of cognate HIV peptide.
(B) Comparison of IFN-γ ELISpot spot forming units (SFU) per well following recognition of peptide-pulsed 721.221, wild-type, and mutant HLA-B*5701-expressing cell lines by polyclonal HIV-specific CD8+ T cells. These data represent the average responses to all three peptides (TW10, KF11, and HW9) across 10 HIV+ individuals. ELISpot values denoted as *** represent those values which are too numerous to count (TNTC). Statistical comparisons were made using a non-parametric Mann-Whitney U test to the 721.221 HLA-B*57 wild-type-expressing control cells. Error bars for (B) indicate standard deviation. Calculated $p$-values were as follows: ****$p < 0.0001$.

that mutation of S70 results in a >50% decrease in binding of HLA-B*5701-TW10 complexes to all cognate TCRs, with one clone having >80% reduced recognition of the S70Q mutant (Figures 6A and 6B). Of note, TW10-specific clone 2 used in these studies (Figure 5) was the same CD8+ T cell clone utilized for the functional assessments of T cell recognition and elimination (Figures 2 and 3).

Structural analysis of the HLA-B*5701 bound by TW10 demonstrates that S70 forms a hydrogen bond with Q7 of the TW10 peptide to maintain the viral epitope in a specific conformation within the HLA binding cleft (Figure 6C). Substitution of S70 with Gln (Q) in HLA-B*0702 results in a loss of this key interaction and potentially a repulsive Gln-Gln interaction (Figure 6D) that likely alters the TW10 epitope conformation that contributes to the decrease in the binding of TW10-specific TCRs to HLA class I-peptide complexes. This is similar to what is observed when S70 is mutated to Lys (K) in both HLA-B*5701-TW10 and HLA-B*5703-KF11 (Figure S3). In contrast, we find that substitution of S70 with Asn (N) preserves the side chain interaction between HLA-B*5701 and Q7 of TW10, which likely explains why it has no effect on HIV-specific CD8+ T cell recognition and elimination (Figures 2, 3, and 4), while mutation to Ala does not lead to repulsive side-chain interactions. These data suggest that residues that occupy the polymorphic position 70 in HLA-B modulate HIV outcomes by maintaining specific conformations of HLA-restricted viral peptides in the HLA binding pocket for interaction with TCRs on HIV-specific CD8+ T cells.

## Effect of residue mutation at polymorphic HLA-B position 97 on KIR interactions with HLA class I-peptide complexes

The lack of impact of mutations to V97 on CD8+ T cell recognition and elimination or HLA class I-peptide stability was unexpected, given that V97 is located in the peptide-binding groove of the HLA-B*5701 molecule and has previously been suggested to mediate HLA class I molecule folding.[42] We therefore compared the previously solved crystal structures of the HLA-B*5701-TW10 peptide complex (PDB: 5V5M)[35] and HLA-B*5801-TW10 peptide complex (PDB: 5V5L),[35] given that HLA-B*5801 is highly conserved in sequence to HLA-B*5701, but has an arginine (R) at position 97 rather than a valine. This revealed a marked difference in the conformation of the TW10 peptide between the two HLA class I alleles, particularly with respect to the TW10 epitope isoleucine residue at position 8 (Ile8), whose buried hydrophobic side chain in HLA-B*5701 becomes highly exposed in HLA-B*5801 (Figure 7A).

Since residues within viral peptides at position 8 are frequently targets of KIR molecules found on natural killer (NK) cells and CD8+ T cells, we evaluated the crystal structure of the HLA-B*5701-TW10-KIR3DL1 complex (PDB: 5T6Z).[43] We were additionally inclined to evaluate this interaction given that the combination of B*5701 and the inhibitory KIR3DL1 molecule (particularly high inhibitory subtypes such as KIR3DL1*001) is associated with improved clinical HIV outcomes.[30] This structural analysis revealed that residues Tyr200 and Ser282 in KIR3DL1 make several water-bridged hydrogen bonds with the main chain atoms of Ile8, Gly9, and Trp10 in the TW10 peptide (Figure 7B). However, when we construct a model using the HLA-B*5801-TW10 crystal structure and overlay it onto the HLA-B*5701-TW10-KIR3DL1 crystal structure, we find that the exposed hydrophobic side chain of Ile8 in the HLA-B*5801-TW10 complex would likely disrupt the water-bridged hydrogen bond network with KIR3DL1 (Figure 7C). This structural analysis therefore suggests that mutation of position 97 could impact HLA class I-peptide-KIR molecule interactions.

To explore this hypothesis in more detail, we first utilized the soluble monomers of wild-type HLA-B*5701 and the V97S mutant in complex with TW10 that we had generated for our thermal denaturation assays and assessed their binding to KIR3DL1-Fc molecules by surface plasmon resonance. Given that Ser97 was associated with relatively higher quantitative viral loads in comparison to Val97, similarly to Arg97,[15] we felt that this was

CellPress
OPEN ACCESS

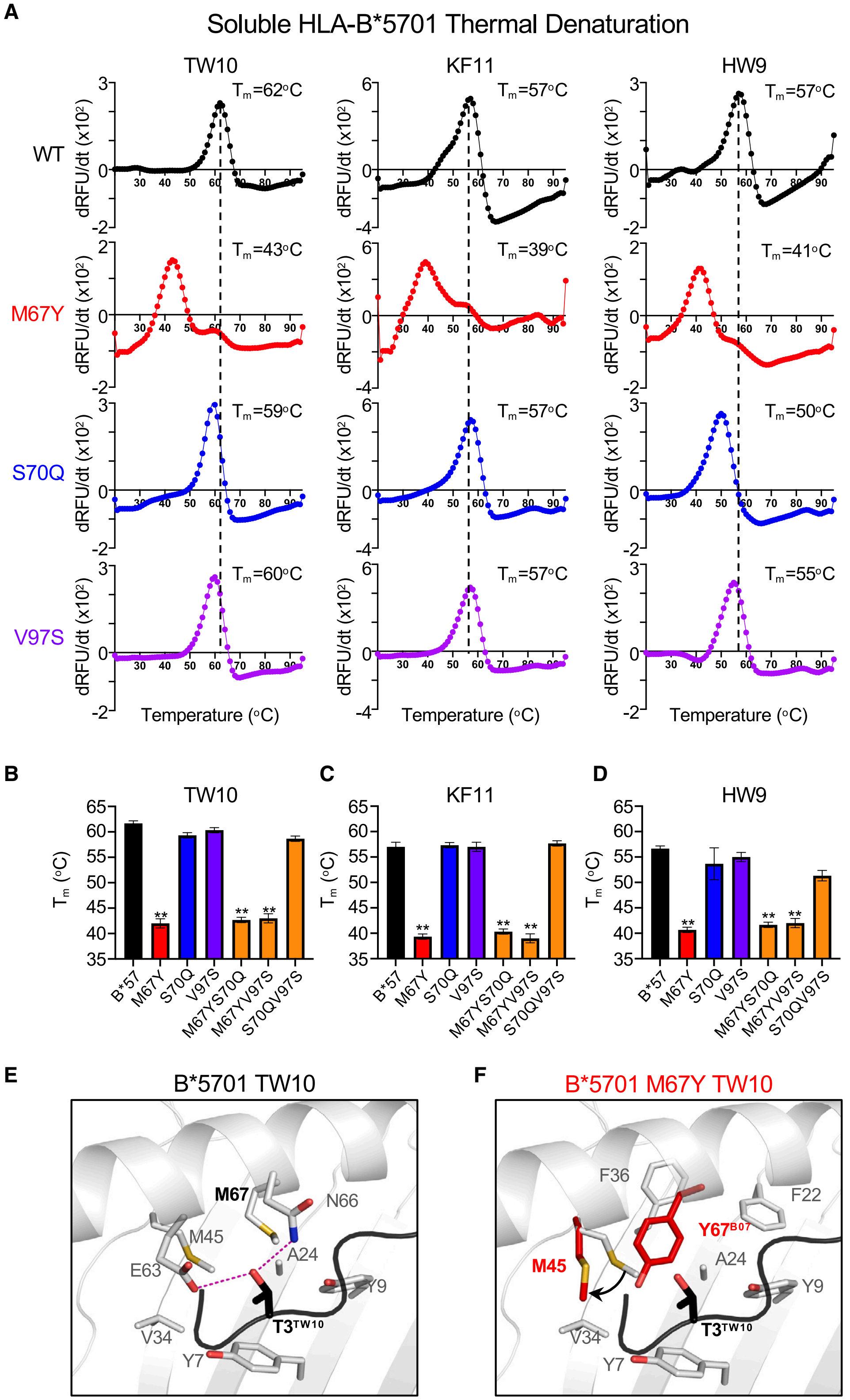


**Figure 5. Mutation of residue 67 affects HLA-B*5701-HIV peptide complex stability**

(A) Representative thermal denaturation of wild-type and mutant HLA-B*5701-peptide monomers for TW10, KF11, and HW9 epitopes. The x axis depicts temperature (20°C–95°C). The y axis depicts the derivative of the temperature versus SYPRO orange dye fluorescence (-dRFU/dT). The thermal stability ($T_m$) is indicated for each wild-type and mutant HLA-B*5701-peptide complex.

*(legend continued on next page)*

suitable for an initial comparison. These studies revealed that the V97S mutation resulted in a ~40% decrease in the dissociation constant ($K_D$) between HLA-B*5701-TW10 and KIR3DL1 (B*5701 wild type $K_D$ = 134μM; B*5701 V97S mutant $K_D$ = 185μM) (Figures 7D and S4). Given that it was somewhat difficult to discern a clear difference due to the low affinity of monomeric HLA class I-KIR molecule interactions, we next generated fluorescently labeled, higher order HLA-B*5701 wild type and V97S dextramers and assessed their avidity to a KIR3DL1-expressing cell line by flow cytometry. This revealed a more substantial and significant difference between wild type and V97S mutant binding to KIR3DL1 (Figures 7E and 7F).

To further confirm the effect of both V97S and V97R mutations on HLA class I-peptide-KIR interactions, we utilized a previously described KIR3DL1 reporter cell line[34] which was stably transduced with a chimeric high inhibitory KIR3DL1*001 receptor linked to the cytoplasmic tail of CD3ζ that triggers expression of CD69 upon binding of a ligand to KIR3DL1, such as HLA-B*5701-peptide complexes (Figure 7G). We therefore co-cultured the reporter cell line with HLA-B*5701 wild type and V97 mutant cell lines (V97S and V97R), pulsed with TW10, KF11, or HW9 peptides, prior to an assessment of the induction of surface CD69 expression (Figure S5). This revealed that both the V97S and V97R HLA-B*5701 mutants had a significant effect on reducing KIR3DL1 reporter cell activation to all three epitopes (Figures 7H–7J). Collectively, these data suggest that position 97 modulates KIR receptor binding in protective HLA class I-peptide complexes.

### Effects of residue mutation at polymorphic HLA-B position 156 on HIV-specific CD8+ T cell recognition and elimination

While we evaluated the role of residues at position 67, 70, and 97 on HLA class I function, a new GWAS study that examined a large multi-ancestry cohort demonstrated that residues at position 156 in *HLA-B* also have a significant impact on HIV control and progression.[15] We therefore first performed a structural analysis of leucine 156 in HLA-B*5701 and found that it maintains a hydrophobic microenvironment with the HLA D-pocket that facilitates the binding and conformation of HIV epitopes within the peptide-binding groove (Figure 8A). To determine the functional contribution of Leu156 (L156), we engineered a mutant HLA-B*5701 expressing cell line by substituting Leu156 (L156) with Arg (L156R), given that Arg was associated with the largest risk effect[15] and is both an established HLA-B*57 micropolymorphism associated with reduced HIV control[44] and present in the risk allele *HLA-B*0702*. We subsequently examined HIV-specific $CD8^+$ T cell recognition of HLA-B*5701 wild type and L156R mutant cells for the TW10, KF11, and HW9 epitopes by IFN-γ ELISpot using cognate epitope-specific $CD8^+$ T cell clones. This revealed that the L156R mutation leads to a significant decrease in HIV-specific $CD8^+$ T cell recognition for the TW10 and KF11 epitopes but not HW9 (Figures 8A and 8B). This was also shown by HIV-specific $CD8^+$ T cell elimination of peptide-pulsed wild type and L156R mutant HLA-B*5701-expressing cells (Figures 8C and 8D).

Similar to our studies of residues at positions 67, 70, and 97, we subsequently evaluated the effect of the L156R mutation on HLA class I-peptide stability, TCR binding, and KIR3DL1 recognition. Thermal denaturation of soluble HLA-B*5701 L156R monomers was associated with significant decreases in $T_m$ for TW10 and KF11, but not HW9 (Figures 8F–8I), highlighting the key role that L156 plays in modulating HLA class I-peptide stability, while also providing a putative mechanistic explanation for the distinct effects of the L156R mutation on epitope recognition by HIV-specific $CD8^+$ T cells (Figures 8B–8E). In addition to the reduced thermostability of the HLA-B*57 L156R mutants bound by TW10 and KF11, we also observed a significant decrease in L156R mutant surface expression relative to the wild-type HLA-B*5701 on transduced 721.221 cells (Figures 8J and 8K). Given that the $T_m$ for the HLA-B*5701-TW10 L156R mutant (51°C) was still within a comparable range to other known immunogenic $CD8^+$ T cell epitopes in HIV,[40] we generated a fluorescently labeled mutant HLA-B*5701-TW10 L156R tetramer and assessed its binding to the three TW10-specific $CD8^+$ T cell clones utilized previously for our analysis of the S70Q mutation. This revealed similarly that there was a significant effect of the L156R mutation on HLA-B*5701-TW10 peptide interactions with cognate TCRs (Figures 8L and 8M). In contrast, there was no significant cumulative effect of L156R on KIR3DL1 recognition of HLA-B*5701-HIV peptide complexes (Figures 8N and 8O).

Structural analysis of L156 to Arg (R) in HLA-B*5701 bound by TW10 (PDB: 5V5M) and B*5703 bound by KF11 (PDB: 2PK) reveals that this mutation (Figure S7) leads to a substantial change in the hydrophobic character of the HLA D-pocket, which likely explains why this substitution significantly affects HLA class I-peptide stability and $CD8^+$ T cell recognition. Collectively, these data illustrate the importance of position 156 in protective HLA class I alleles in mediating stable interactions between HIV peptides and HLA class I molecules and the subsequent interaction of HLA-peptide complexes with TCRs.

## DISCUSSION

Elucidating mechanisms by which HLA-B modulates outcomes of HIV infection will greatly assist in our understanding of how to achieve a functional HIV cure. Toward this objective, we systematically assessed the role of specific amino acid residues at positions 67, 70, 97, and 156 which have been shown by immunogenetic studies to be responsible for the impact of HLA-B on HIV infection and collectively are more strongly associated with HIV control than any individual allele.[12,15] This provided

(B) Comparison of average $T_m$s for wild-type, single and double HLA-B*5701 mutants for the TW10, (C) KF11 and (D) HW9 epitopes. Statistical comparisons were made using an unpaired t test to the soluble wild-type HLA-B*57-HIV peptide monomer control. Error bars for (B–D) indicate standard deviation from six biological replicates performed independently. Calculated $p$-values were as follows: $^{**}p < 0.01$.
(E) Structural analysis of the wild-type HLA-B*5701-TW10 complex and (F) HLA-B*5701 M67Y *in silico* mutant model presenting TW10. HLA-B*5701 is shown as gray ribbon and stick, while peptides were shown in black ribbon and stick. The model of the HLA-B*5701 M67Y mutation (red) was constructed using the previously solved crystal structure of HLA-B*0702 (PDB: 5EO0) to overlay the naturally occurring Tyr at position 67 in B*0702 onto the crystal structure of the HLA-B*5701-TW10 complex (PBD: 5V5M).

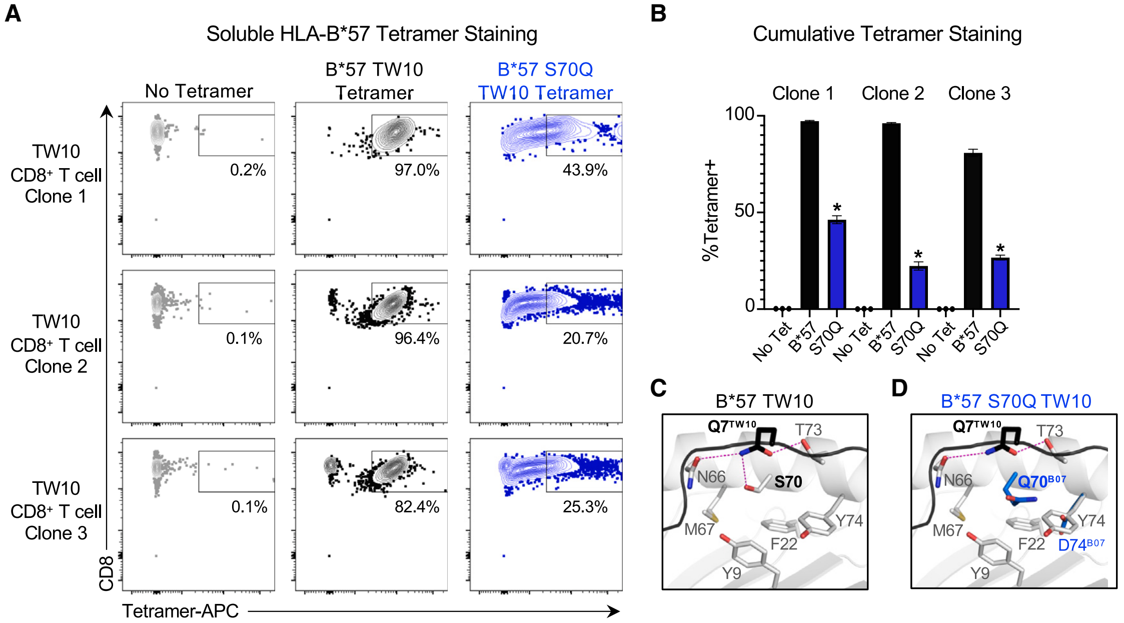


**Figure 6. Mutation of residue 70 affects interactions between HLA-B*5701-peptide complexes and HIV-specific TCRs**

(A) Binding of APC-labeled wild-type and S70Q HLA-B*5701-TW10 tetramers to three distinct TW10-specific CD8$^+$ T cell clones.

(B) Comparison of %Tetramer$^+$ TW10-specific CD8$^+$ T cell clones following incubation with no tetramer, wild-type HLA-B*5701-TW10 tetramer or HLA-B*5701-TW10 S70Q mutant tetramer. Statistical comparisons were made using an unpaired t test to the B*5701 tetramer control. Error bars from (B) indicate standard deviation from three biological replicates performed independently. Calculated *p*-values were as follows: $^*p < 0.05$.

(C) Structural analysis of the wild-type HLA-B*5701-TW10 complex and (D) HLA-B*5701 S70Q *in silico* mutant model presenting TW10. HLA-B*5701 is shown as gray ribbon and stick, while peptides were shown in black ribbon and stick. The model of the HLA-B*5701 S70Q mutation (blue) was constructed using the previously solved crystal structures of HLA-B*0702 (PDB: 5EO0) to overlay the Gln that naturally occurs at position 70 with B*0702 onto the crystal structure of the HLA-B*5701-TW10 complex (PBD: 5V5M).

a clear reductionist opportunity to delineate mechanisms of HLA-associated HIV control. We assessed these residues in HLA-B*5701, a model protective HLA class I allele,[6,11,12] and demonstrated that mutation of M67, S70, V97, and L156 distinctly affect either the stability of HLA class I-HIV peptide complexes, the recognition by HIV-specific CD8$^+$ T cell TCRs or notably for V97, the binding of HLA class I-peptide complexes to KIR molecules. These findings therefore provide a functional and structural explanation for causal insights from GWAS studies to demonstrate that modulation of the stability and conformation of viral peptides that bind to specific HLA-B alleles for recognition by both TCR and KIR molecules is the molecular and genetic basis by which HLA-B impacts outcomes to HIV infection. Given that HLA-B alleles greatly differ in terms of the epitopes that engage their distinct peptide-binding grooves, these data further indicate that epitope specificity is a key component of HIV control and also highlight the relative contribution of HLA-peptide-KIR interactions.

While HLA-B*5701 has been shown to be the strongest HLA determinant of low viral loads, the expression of B*5701 is neither necessary nor sufficient for viral control. Rather what appears to distinguish individuals who successfully control HIV, independent of HLA haplotype, is the presence of highly proliferative and functional CD8$^+$ T cell responses directed toward epitopes derived from structurally constrained (“networked”) regions of the HIV proteome.[20,45–47] In addition, it has been observed that protective HLA class I alleles, such as *B*5701*, are more likely to present and be durably stabilized by epitopes derived from networked regions,[20] which is contrast to risk alleles that are more likely to present epitopes from structurally flexible regions vulnerable to mutational escape. Such data have clearly indicated that epitope specificity is a key component of HIV control. Importantly, the findings in this study now show in an orthogonal manner that the polymorphic HLA-B residues identified by immunogenetics studies specifically affect the stability and conformation of networked B*5701-restricted HIV epitopes (KF11, TW10, and HW9) and their subsequent recognition by HIV-specific CD8$^+$ T cells and KIR molecules. In light of recent work demonstrating that protective HLA class I alleles are also preferentially stabilized by networked epitopes,[40] it is likely that residues at positions 67, 70, 97, and 156 in HLA-B directly impact the diversity of peptides that bind to distinct HLA-B alleles. In the case of the protective HLA-B*5701 allele, M67, S70, V97, and L156 facilitate the preferential presentation of highly networked epitopes,[40] leading to improved control of HIV.[20] In contrast, the risk allele HLA-B*0702 expresses Y67, Q70, S97, and R156, which leads to substantially reduced stabilization of highly networked epitopes[40] and decreased binding of HLA-B*5701 to networked epitopes when these mutations are introduced. Thus, these specific polymorphic residues appear to mediate distinct outcomes to HIV infection by modulating the stabilization and presentation of specific epitopes.

While certain mutations of M67, S70, V97, and L156 modulate HLA-B*5701 function, not all evaluated substitutions had a

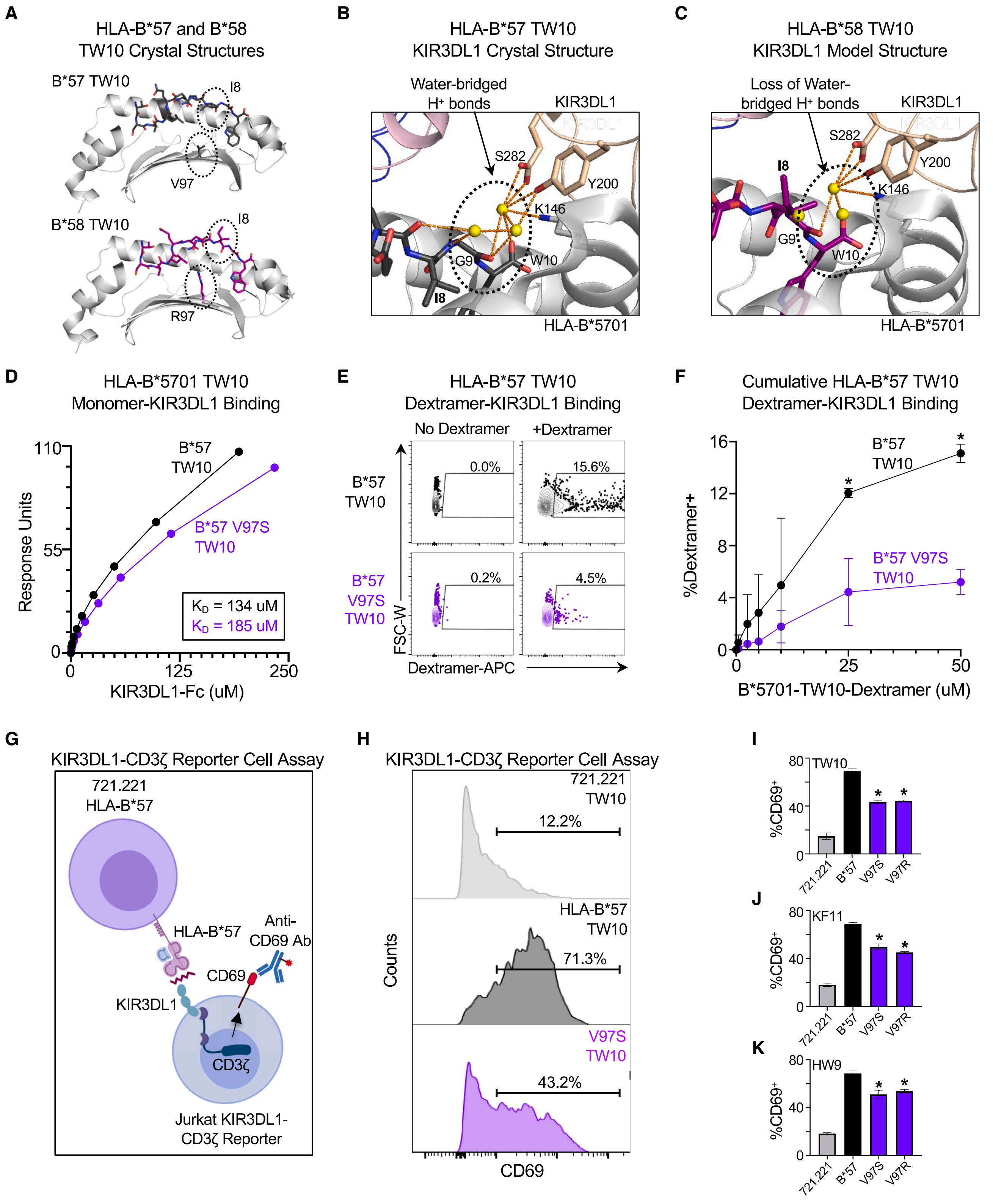


**Figure 7. Mutation of residue 97 affects interactions between HLA-B*5701-peptide complexes and KIR3DL1**

(A) Comparison of TW10 peptide orientation in the previously solved HLA-B*5701-TW10 crystal structure (PDB: 5V5M) and HLA-B*5801-TW10 crystal structure (PDB: 5V5L).

(legend continued on next page)

detrimental effect, particularly for M67 and S70 (i.e., M67F, M67A, S70N, and S70A). Structural analyses of the HLA-B*5701-TW10 and HLA-B*5703-KF11 peptide complexes demonstrated that these differences were due to either the relative impact of residue substitutions on amino acid microenvironments within the HLA-peptide binding pocket or their impact on interactions between HIV-specific TCRs and HLA class I-HIV peptide complexes. We specifically focused on mutations that were naturally found in other HLA class I allelic variants, as this afforded the opportunity to place any biochemical and immunologic insights regarding these amino acid residues within the context of previously established impact on quantitative viral loads. It is therefore notable that residues associated with the highest relative viral load (Y at position 67, Q at position 70)[12] had the most substantial effect on HIV-specific CD8$^+$ T cell recognition and elimination when introduced as substitutions in HLA-B*5701.

We made a similar observation for the Arg mutation at position L156, which apart from having the highest associated risk effect,[15] is also expressed as a B*57 micropolymorphism in the *HLA-B*5702* allele. Interestingly, individuals who express B*5702 have substantially reduced frequencies of CD8$^+$ T cell responses to the protective TW10 and KF11 epitopes and significantly higher viral load set points in comparison to other protective B*57 alleles that have a Leu at position 156 (i.e., *B*5701* and *B*5703*).[44] The findings reported here provide a mechanistic basis for these published observations as the L156R mutation clearly has a strong impact on the HLA-B*5701-peptide stability for both epitopes and may also contribute by additionally disrupting TCR-based recognition of the TW10 epitope.

The lack of effect of V97 mutations on HIV-specific CD8$^+$ T cell recognition was surprising, given that it has been identified as the residue with the strongest association with HIV control.[12,13,15,48] Importantly, we demonstrate that mutation of V97 disrupts interactions between HLA-B*5701-HIV peptide complexes and KIR3DL1 molecules, further underscoring the importance of HLA-KIR interactions in modulating HIV outcomes. This observation builds on prior work that identified an innate partnership between HLA-Bw4 molecules and high inhibitory KIR3DL1 subtypes (such as KIR3DL1*001) in slowing HIV progression,[30] given that strong inhibitory KIR3DL1 capacity is critical for NK cell development to quench autoreactivity[49,50] and is subsequently associated with greater NK cell responsiveness during viral infections.[51,52] In fact, weak or missing inhibitory signals result in poor activating potential by NK cells,[53] which further implicates the role of V97 and NK cells expressing inhibitory KIR molecules in HIV control. However, in addition, recent insights also suggest that expression of inhibitory KIR molecules on CD8$^+$ T cell enhances their survival and persistence, leading to better outcomes for chronic viral infections, such as HIV.[33] Thus, this potential dual effect that V97 has on both innate and cellular immunity may explain why it is a key residue in determining HLA-mediated HIV outcomes across several immunogenetic studies.[12,15,16]

Additional posited mechanisms of variation of HLA-mediated HIV outcomes involving functional TCR avidity[25–27] and interactions with LILRB2[28] do not have the same genetic or structural basis as HLA-peptide stability and the conformation of peptides to TCR and KIR molecules in the context of the four key polymorphic residues identified by GWAS. While TCRs directed against B*5701-restricted epitopes are conserved,[48] primarily public[54,55] and similar in clonal composition between progressors and controllers,[56,57] differences in TCR ligation can exist between alleles that display epitopes in structurally conserved formats.[41] Importantly, the HLA-B residues that govern these differences are found at positions 114 and 116, which have not been consistently identified as key mediators by GWAS. With respect to LILRB2, this molecule engages HLA class I at the highly conserved $\alpha$3 domain[58] which has similarly not been identified as a differentiating feature between protective and risk HLA-B alleles and also has been shown to not vary significantly with HLA polymorphism.[59]

In summary, our data reveal how the polymorphic residues within HLA-B that are responsible for the impact of certain HLA-B alleles on outcomes from HIV infection mechanistically affect HIV-specific CD8$^+$ T cell recognition and HLA-peptide-KIR interactions. While these residues affect HLA-B molecule function through distinct mechanisms, they collectively establish that modulation of the stability and conformation of viral epitopes within the HLA binding pocket for recognition by TCR and KIR molecules is the molecular and genetic basis by which the host immune response impacts HIV outcomes. In addition, they reveal that KIR molecules potentially play a relatively more prominent role than previously appreciated, given the modulation of their binding to HLA by mutation of residues at position 97, which

(B) Delineation of water-bridged hydrogen bond network between residues in KIR3DL1 (Y200, S282) and the main chain atoms of the TW10 peptide (I8, G9) in the HLA-B*5701-KIR3DL1 crystal structure (PDB: 5T6Z).

(C) Model structure in which the HLA-B*5801-TW10 structure (PDB: 5V5L) is overlayed onto the HLA-B*5701-TW10-KIR3DL1 crystal structure (PDB: 5T6Z) to illustrate disruption of the water-bridged hydrogen bond network between residues in KIR3DL1 (Y200, S282) and the TW10 peptide (I8, G9) due to the change in the orientation of I8 hydrophobic side chain.

(D) Surface plasmon resonance response units (RU) of wild-type (black) HLA-B*5701 TW10 monomer or V97S mutant (purple) binding to immobilized KIR3DL1-Fc in the presence of increasing micromolar concentrations of soluble HLA-B*5701-peptide monomer. These data are a composite of a single replicate (Figure S4).

(E) Representative flow plots of APC-labeled HLA-B*5701-TW10 wild-type or mutant V97S dextramers binding to KIR3DL1*001$^+$ Jurkat cells.

(F) Percentage of KIR3DL1*001$^+$ Jurkat cells bound by increasing micromolar concentrations of APC-labeled wild-type or mutant V97S HLA-B*5701-TW10 dextramer.

(G) Schematic of KIR3DL1-CD3$\zeta$ reporter cell assay. Peptide-pulsed HLA-null 721.221 cells, HLA-B*5701 wild type or mutant cell lines were co-cultured with KIR3DL1-CD3$\zeta$ Jurkat cells prior to assessment of CD69 expression on CD3$^+$ KIR3DL1*001$^+$ Jurkat cells (see Figure S2).

(H) Representative flow histograms of CD69 expression on CD3$^+$ KIR3DL1*001$^+$ Jurkat cells following co-culture with HLA-null 721.221 cells, HLA-B*5701 cells or mutant V97S cells pulsed with TW10 peptide.

(I) Comparison of %CD69$^+$ CD3$^+$ KIR3DL1*001$^+$ Jurkat cells following co-culture with 721.221, wild-type HLA-B*5701 or V97 mutant HLA-B*5701-expressing cell lines pulsed with TW10 peptide, (J) KF11 peptide or (K) HW9 peptide. Statistical comparisons were made using an unpaired t test to either wild-type HLA-B*5701-TW10 monomer, wild-type HLA-B*5701 dextramer, or 721.221 HLA-B*57 wild-type-expressing control cells. Error bars for (I–K) indicate standard deviation from three biological replicates performed independently. Calculated $p$-values were as follows: $*p < 0.05$.

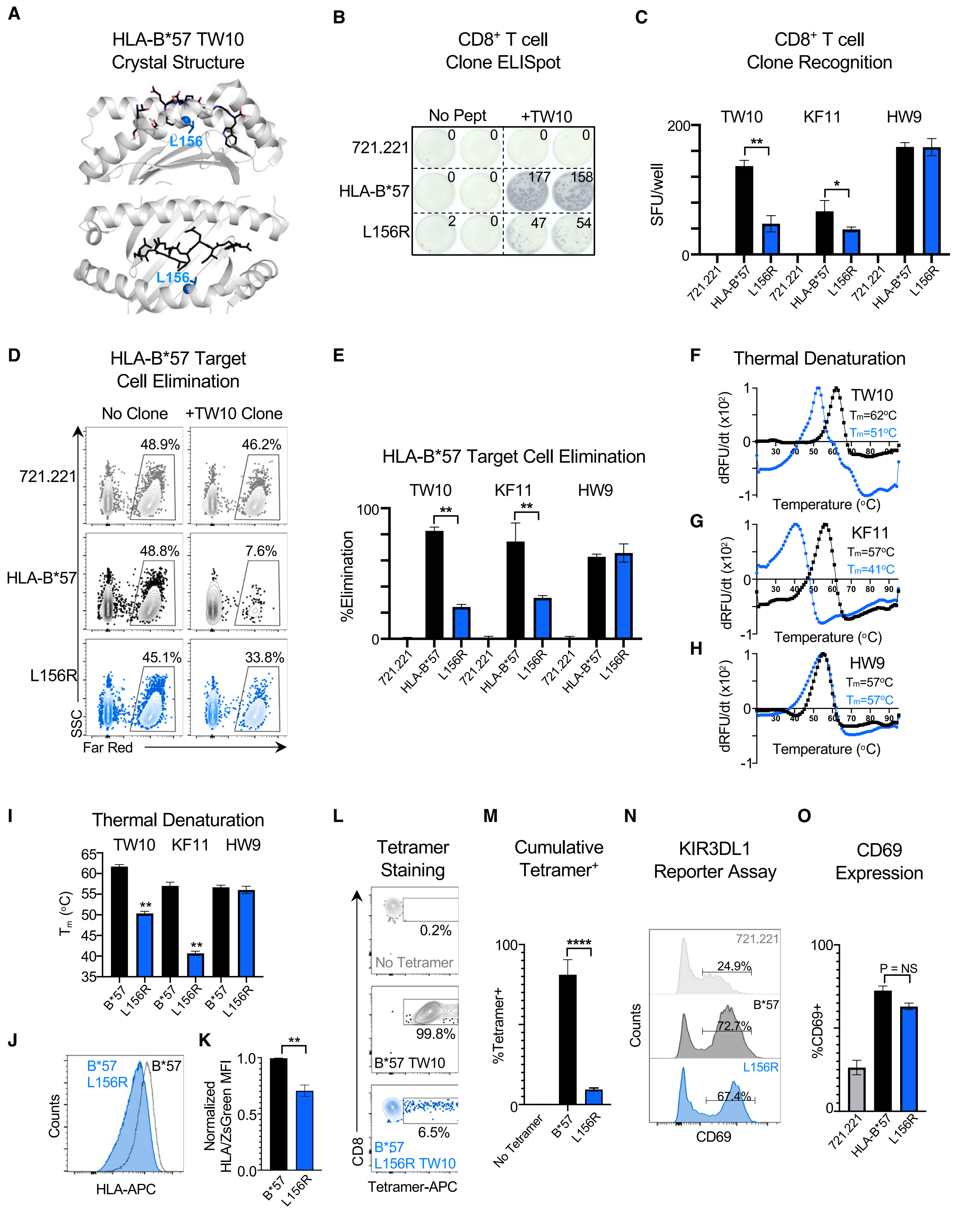


**Figure 8. Mutation of residue 156 affects HLA-B*5701-peptide stability and its interaction with HIV-specific TCRs**

(A) The overall and top-down view of the previously solved three-dimensional structure of HLA-B*5701 bound to the TW10 (Gag p24$_{108-117}$) peptide (PDB: 5V5M). The highlighted Leu 156 residue (light blue), which is present within the peptide-binding groove, shown as a sphere with its residue side chain.

*(legend continued on next page)*

CellPress
OPEN ACCESS

is most strongly associated with viral loads and HIV control.[12,15,16,31] Given that a key differentiating factor of protective and risk HLA-B alleles is the distinct set of epitopes that each HLA class I allele presents, such data further indicate that replicating HIV control will benefit from the selection of specific epitopes which will induce durable CD8+ T cell responses and NK cell immunity, ideally derived from structurally and mutationally constrained regions of the viral proteome.

## STAR★METHODS

Detailed methods are provided in the online version of this paper and include the following:


- KEY RESOURCES TABLE
- RESOURCE AVAILABILITY
  - Lead contact
  - Materials availability
  - Data and code availability
- EXPERIMENTAL MODEL AND STUDY PARTICIPANT DETAILS
  - Cell lines
  - HLA-B*5701 wild-type and mutant-expressing cell line generation
  - HIV-specific CD8+ T cell clone generation
  - Study participants
- METHOD DETAILS
  - Recombinant DNA constructs
  - Site-directed mutagenesis
  - Isolation of primary CD8+ T cells from HIV+ individuals
  - Peptide synthesis reagents
  - Peptide synthesis and analysis
  - CD8+ T cell recognition assay
  - CD8+ T cell elimination assay
  - Soluble HLA class I-peptide monomer expression and purification
  - Thermal denaturation assay
  - Structural analysis of HLA-B*5701-peptide complexes
  - Generation of fluorescently labeled HLA-B*5701-peptide tetramers
  - KIR3DL1 surface plasmon resonance
  - Dextramer assembly
  - KIR3DL1 reporter cell assay
- QUANTIFICATION AND STATISTICAL ANALYSIS


## SUPPLEMENTAL INFORMATION

Supplemental information can be found online at https://doi.org/10.1016/j.str.2024.04.015.

## ACKNOWLEDGMENTS

We thank Shiv Pillai and Charles Crain for their helpful comments on this manuscript. We also thank Wilfredo Garcia-Beltran for providing us with the Jurkat-KIR3DL1*001-CD3ζ reporter cells. This study was supported by NIH grants UM1AI144462 (B.D.W. and G.D.G.), UM1AI164560 (G.D.G.), DP2AI154421 (G.D.G.), R01AI176533 (G.D.G), and DP1DA058476 (G.D.G.). Additional support was provided by the Howard Hughes Medical Institute (B.D.W.), the Ragon Institute of MGH, MIT and Harvard (B.D.W. and G.D.G.), the Mark and Lisa Schwartz Foundation and Enid Schwartz (B.D.W.), and Sandy and Paul Edgerly. G.D.G. is supported by the Bill and Melinda Gates Foundation, a Burroughs Wellcome Career Award for Medical Scientists and Howard Goodman Fellowship. This project has been funded in whole or in part with federal funds from the Frederick National Laboratory for Cancer Research, under contract no. 75N91019D00024. The content of this publication does not necessarily reflect the views or policies of the Department of Health and Human Services, nor does mention of trade names, commercial products, or organizations imply endorsement by the U.S. Government. This Research was supported in part by the Intramural Research Program of the NIH, Frederick National Lab, Center for Cancer Research.

## AUTHOR CONTRIBUTIONS

Conceptualization, R.T.M. and G.D.G.; methodology and investigation, R.T.M., N.K.S., I.M., X.L., M.V.M., C.K., A.Z., F.O., L.M., A.P.T., A.B., and G.D.G.; writing – original draft, G.D.G.; writing – final draft, R.T.M., N.K.S., I.M., X.L., M.C., B.D.W., and G.D.G.; funding acquisition, M.B., M.C., B.D.W., and G.D.G.; supervision, M.B., M.C., B.D.W., and G.D.G.

## DECLARATION OF INTERESTS

B.D.W. and G.D.G have filed patent application PCT/US2020/022403. G.D.G. also reports research funding from Moderna and Merck.

Received: September 26, 2023
Revised: February 27, 2024
Accepted: April 16, 2024
Published: May 10, 2024

(B) Representative IFN-γ ELISpot data of TW10-specific CD8+ T cell clone recognition of HLA-null 721.221 cells, wild-type B*5701-expressing 721.221 cells and mutant L156R B*5701-expressing 721.221 cells incubated in the presence and absence of TW10 peptide.
(C) Comparison of IFN-γ ELISpot spot forming units (SFU) per well following recognition of peptide-pulsed 721.221, wild-type and mutant L156R HLA-B*5701-expressing cell lines by TW10-specific, KF11-specific and HW9-specific CD8+ T cell clones.
(D) Representative CD8+ T cell elimination of peptide-pulsed HLA-null 721.221, HLA-B*5701 wild type, and L156R mutant following co-culture with and without a TW10-specific CD8+ T cell clone.
(E) Comparison of %elimination of peptide-loaded 721.221, wild-type and mutant L156R HLA-B*5701-expressing cell lines by TW10-specific, KF11-specific and HW9-specific CD8+ T cell clones.
(F) Thermal denaturation of wild-type and mutant L156R HLA-B*5701-peptide monomers for TW10, (G) KF11 and (H) HW9 epitopes. The x axis depicts temperature (20°C–95°C). The y axis depicts the derivative of the temperature versus fluorescence (-dRFU/dT). The thermal stability ($T_m$) is indicated for each wild-type and mutant L156R HLA-B*5701-peptide complex.
(I) Comparison of average $T_m$s for wild type and L156R HLA-B*5701 mutants for the TW10, KF11, and HW9 epitopes. Statistical comparisons were made using an unpaired t test to the soluble wild-type HLA-B*57-HIV peptide monomer control. Each experiment was performed three times independently.
(J) Representative wild-type (open dashed) and L156R mutant (filled blue) HLA-B*5701 expression in transduced 721.221 cells.
(K) Ratio of surface HLA-B*5701 expression to ZsGreen transgene expression for B*5701 L156R mutant normalized to wild-type HLA-B*5701-expressing cell line (black).
(L) Representative binding of APC-labeled wild-type and L156R HLA-B*5701-TW10 tetramers to a TW10-specific CD8+ T cell clone.
(M) Comparison of %Tetramer+ TW10-specific CD8+ T cell clones following incubation with no tetramer, wild-type HLA-B*5701-TW10 tetramer, or HLA-B*5701-TW10 L156R mutant tetramer.
(N) Representative flow histograms of CD69 expression on CD3+ KIR3DL1*001+ Jurkat cells following co-culture with HLA-null 721.221 cells, HLA-B*5701 cells, or mutant L156R cells pulsed with TW10 peptide.
(O) Comparison of average %CD69+ CD3+ KIR3DL1*001+ Jurkat cells following co-culture with 721.221, wild-type HLA-B*5701, or L156R mutant HLA-B*5701-expressing cell lines pulsed with TW10, KF11, and HW9 peptides. Statistical comparisons were made using an unpaired t test to either 721.221 HLA-B*57 wild-type-expressing control cells or soluble wild-type HLA-B*57-HIV peptide monomer control. Error bars for (C), (E), (I), (K), (M), and (O) indicate standard deviation from three biological replicates performed independently. Calculated $p$-values were as follows: *$p < 0.05$, **$p < 0.01$, ****$p < 0.0001$.

## REFERENCES


1. O'Brien, T.R., Blattner, W.A., Waters, D., Eyster, E., Hilgartner, M.W., Cohen, A.R., Luban, N., Hatzakis, A., Aledort, L.M., Rosenberg, P.S., et al. (1996). Serum HIV-1 RNA levels and time to development of AIDS in the Multicenter Hemophilia Cohort Study. JAMA *276*, 105–110.
2. Quinn, T.C., Wawer, M.J., Sewankambo, N., Serwadda, D., Li, C., Wabwire-Mangen, F., Meehan, M.O., Lutalo, T., and Gray, R.H. (2000). Viral Load and Heterosexual Transmission of Human Immunodeficiency Virus Type 1. N. Engl. J. Med. *342*, 921–929.
3. Gray, R.H., Wawer, M.J., Brookmeyer, R., Sewankambo, N.K., Serwadda, D., Wabwire-Mangen, F., Lutalo, T., Li, X., vanCott, T., and Quinn, T.C.; Rakai Project Team (2001). Probability of HIV-1 transmission per coital act in monogamous, heterosexual, HIV-1-discordant couples in Rakai, Uganda. Lancet *357*, 1149–1153.
4. Collins, D.R., Gaiha, G.D., and Walker, B.D. (2020). CD8+ T cells in HIV control, cure and prevention. Nat. Rev. Immunol. *20*, 471–482. https://doi.org/10.1038/s41577-020-0274-9.
5. Kaslow, R.A., Carrington, M., Apple, R., Park, L., Muñoz, A., Saah, A.J., Goedert, J.J., Winkler, C., O'Brien, S.J., Rinaldo, C., et al. (1996). Influence of combinations of human major histocompatibility complex genes on the course of HIV–1 infection. Nat. Med. *2*, 405–411.
6. Migueles, S.A., Sabbaghian, M.S., Shupert, W.L., Bettinotti, M.P., Marincola, F.M., Martino, L., Hallahan, C.W., Selig, S.M., Schwartz, D., Sullivan, J., and Connors, M. (2000). HLA B* 5701 is highly associated with restriction of virus replication in a subgroup of HIV-infected long term nonprogressors. Proc. Natl. Acad. Sci. USA *97*, 2709–2714.
7. Carrington, M., Nelson, G.W., Martin, M.P., Kissner, T., Vlahov, D., Goedert, J.J., Kaslow, R., Buchbinder, S., Hoots, K., and O'Brien, S.J. (1999). HLA and HIV-1: Heterozygote Advantage and B*35-Cw*04 Disadvantage. Science *283*, 1748–1752.
8. Gao, X., Nelson, G.W., Karacki, P., Martin, M.P., Phair, J., Kaslow, R., Goedert, J.J., Buchbinder, S., Hoots, K., Vlahov, D., et al. (2001). Effect of a Single Amino Acid Change in MHC Class I Molecules on the Rate of Progression to AIDS. N. Engl. J. Med. *344*, 1668–1675.
9. Li, S.S., Hickey, A., Shangguan, S., Ehrenberg, P.K., Geretz, A., Butler, L., Kundu, G., Apps, R., Creegan, M., Clifford, R.J., et al. (2022). HLA-B*46 associates with rapid HIV disease progression in Asian cohorts and prominent differences in NK cell phenotype. Cell Host Microbe *30*, 1173–1185.e8.
10. Kiepiela, P., Leslie, A.J., Honeyborne, I., Ramduth, D., Thobakgale, C., Chetty, S., Rathnavalu, P., Moore, C., Pfafferott, K.J., Hilton, L., et al. (2004). Dominant influence of HLA-B in mediating the potential co-evolution of HIV and HLA. Nature *432*, 769–775.
11. Fellay, J., Shianna, K.V., Ge, D., Colombo, S., Ledergerber, B., Weale, M., Zhang, K., Gumbs, C., Castagna, A., Cossarizza, A., et al. (2007). A whole-genome association study of major determinants for host control of HIV-1. Science *317*, 944–947.
12. International HIV Controllers Study, Pereyra, F., Jia, X., McLaren, P.J., Telenti, A., de Bakker, P.I.W., Walker, B.D., Ripke, S., Brumme, C.J., Pulit, S.L., et al. (2010). The major genetic determinants of HIV-1 control affect HLA class I peptide presentation. Science *330*, 1551–1557.
13. McLaren, P.J., Coulonges, C., Bartha, I., Lenz, T.L., Deutsch, A.J., Bashirova, A., Buchbinder, S., Carrington, M.N., Cossarizza, A., Dalmau, J., et al. (2015). Polymorphisms of large effect explain the majority of the host genetic contribution to variation of HIV-1 virus load. Proc. Natl. Acad. Sci. USA *112*, 14658–14663.
14. McLaren, P.J., Pulit, S.L., Gurdasani, D., Bartha, I., Shea, P.R., Pomilla, C., Gupta, N., Gkrania-Klotsas, E., Young, E.H., Bannert, N., et al. (2017). Evaluating the Impact of Functional Genetic Variation on HIV-1 Control. J. Infect. Dis. *216*, 1063–1069.
15. Luo, Y., Kanai, M., Choi, W., Li, X., Sakaue, S., Yamamoto, K., Ogawa, K., Gutierrez-Arcelus, M., Gregersen, P.K., Stuart, P.E., et al. (2021). A high-resolution HLA reference panel capturing global population diversity enables multi-ancestry fine-mapping in HIV host response. Nat. Genet. *53*, 1504–1516.
16. McLaren, P.J., Porreca, I., Iaconis, G., Mok, H.P., Mukhopadhyay, S., Karakoc, E., Cristinelli, S., Pomilla, C., Bartha, I., Thorball, C.W., et al. (2023). Africa-specific human genetic variation near CHD1L associates with HIV-1 load. Nature *620*, 1025–1030. https://doi.org/10.1038/s41586-023-06370-4.
17. Goulder, P.J., Bunce, M., Krausa, P., McIntyre, K., Crowley, S., Morgan, B., Edwards, A., Giangrande, P., Phillips, R.E., and McMichael, A.J. (1996). Novel, cross-restricted, conserved, and immunodominant cytotoxic T lymphocyte epitopes in slow progressors in HIV type 1 infection. AIDS Res. Hum. Retrovir. *12*, 1691–1698.
18. Dahirel, V., Shekhar, K., Pereyra, F., Miura, T., Artyomov, M., Talsania, S., Allen, T.M., Altfeld, M., Carrington, M., Irvine, D.J., et al. (2011). Coordinate linkage of HIV evolution reveals regions of immunological vulnerability. Proc. Natl. Acad. Sci. USA *108*, 11530–11535.
19. Ferguson, A.L., Mann, J.K., Omarjee, S., Ndung'u, T., Walker, B.D., and Chakraborty, A.K. (2013). Translating HIV sequences into quantitative fitness landscapes predicts viral vulnerabilities for rational immunogen design. Immunity *38*, 606–617.
20. Gaiha, G.D., Rossin, E.J., Urbach, J., Landeros, C., Collins, D.R., Nwonu, C., Muzhingi, I., Anahtar, M.N., Waring, O.M., Piechocka-Trocha, A., et al. (2019). Structural topology defines protective CD8+ T cell epitopes in the HIV proteome. Science *364*, 480–484.
21. McMichael, A.J., and Carrington, M. (2019). Topological perspective on HIV escape. Science *364*, 438–439.
22. Kosmrlj, A., Read, E.L., Qi, Y., Allen, T.M., Altfeld, M., Deeks, S.G., Pereyra, F., Carrington, M., Walker, B.D., and Chakraborty, A.K. (2010). Effects of thymic selection of the T-cell repertoire on HLA class I-associated control of HIV infection. Nature *465*, 350–354.
23. Pohlmeyer, C.W., Buckheit, R.W., 3rd, Siliciano, R.F., and Blankson, J.N. (2013). CD8+ T cells from HLA-B*57 elite suppressors effectively suppress replication of HIV-1 escape mutants. Retrovirology *10*, 152.
24. Li, X., Singh, N.K., Collins, D.R., Ng, R., Zhang, A., Lamothe-Molina, P.A., Shahinian, P., Xu, S., Tan, K., Piechocka-Trocha, A., et al. (2023). Molecular basis of differential HLA class I-restricted T cell recognition of a highly networked HIV peptide. Nat. Commun. *14*, 2929.
25. Berger, C.T., Frahm, N., Price, D.A., Mothe, B., Ghebremichael, M., Hartman, K.L., Henry, L.M., Brenchley, J.M., Ruff, L.E., Venturi, V., et al. (2011). High-functional-avidity cytotoxic T lymphocyte responses to HLA-B-restricted Gag-derived epitopes associated with relative HIV control. J. Virol. *85*, 9334–9345.
26. Appay, V., and Iglesias, M.C. (2011). Antigen sensitivity and T-cell receptor avidity as critical determinants of HIV control. Curr. Opin. HIV AIDS *6*, 157–162.
27. Chen, H., Ndhlovu, Z.M., Liu, D., Porter, L.C., Fang, J.W., Darko, S., Brockman, M.A., Miura, T., Brumme, Z.L., Schneidewind, A., et al. (2012). TCR clonotypes modulate the protective effect of HLA class I molecules in HIV-1 infection. Nat. Immunol. *13*, 691–700.
28. Bashirova, A.A., Martin-Gayo, E., Jones, D.C., Qi, Y., Apps, R., Gao, X., Burke, P.S., Taylor, C.J., Rogich, J., Wolinsky, S., et al. (2014). LILRB2 interaction with HLA class I correlates with control of HIV-1 infection. PLoS Genet. *10*, e1004196.
29. Martin, M.P., Gao, X., Lee, J.-H., Nelson, G.W., Detels, R., Goedert, J.J., Buchbinder, S., Hoots, K., Vlahov, D., Trowsdale, J., et al. (2002). Epistatic interaction between KIR3DS1 and HLA-B delays the progression to AIDS. Nat. Genet. *31*, 429–434.
30. Martin, M.P., Qi, Y., Gao, X., Yamada, E., Martin, J.N., Pereyra, F., Colombo, S., Brown, E.E., Shupert, W.L., Phair, J., et al. (2007). Innate partnership of HLA-B and KIR3DL1 subtypes against HIV-1. Nat. Genet. *39*, 733–740.
31. Vaidya, S.A., Streeck, H., Pereyra, F., Rosenberg, E.S., Walker, B.D., and Altfeld, M. (2012). The viral set point in primary HIV infection is associated

CellPress
OPEN ACCESS

with specific amino acids in position 97 of MHC class I. Retrovirology *9*, P163.

32. Martin, M.P., Naranbhai, V., Shea, P.R., Qi, Y., Ramsuran, V., Vince, N., Gao, X., Thomas, R., Brumme, Z.L., Carlson, J.M., et al. (2018). Killer cell immunoglobulin–like receptor 3DL1 variation modifies HLA-B*57 protection against HIV-1. J. Clin. Invest. *128*, 1903–1912.
33. Boelen, L., Debebe, B., Silveira, M., Salam, A., Makinde, J., Roberts, C.H., Wang, E.C.Y., Frater, J., Gilmour, J., Twigger, K., et al. (2018). Inhibitory killer cell immunoglobulin-like receptors strengthen CD8+ T cell-mediated control of HIV-1, HCV, and HTLV-1. Sci. Immunol. *3*, eaao2892. https://doi.org/10.1126/sciimmunol.aao2892.
34. Salzberger, W., Garcia-Beltran, W.F., Dugan, H., Gubbala, S., Simoneau, C., Gressens, S.B., Jost, S., and Altfeld, M. (2015). Influence of glycosylation inhibition on the binding of KIR3DL1 to HLA-B*57:01. PLoS One *10*, e0145324.
35. Li, X., Lamothe, P.A., Walker, B.D., and Wang, J.-H. (2017). Crystal structure of HLA-B* 5801 with a TW10 HIV Gag epitope reveals a novel mode of peptide presentation. Cell. Mol. Immunol. *14*, 631–634.
36. Parham, P., Barnstable, C.J., and Bodmer, W.F. (1979). Use of a monoclonal antibody (W6/32) in structural studies of HLA-A, B, C antigens. J. Immunol. *123*, 342–349.
37. Navis, M., Schellens, I.M.M., van Swieten, P., Borghans, J.A.M., Miedema, F., Kootstra, N.A., van Baarle, D., and Schuitemaker, H. (2008). A nonprogressive clinical course in HIV-infected individuals expressing human leukocyte antigen B57/5801 is associated with preserved CD8+ T lymphocyte responsiveness to the HW9 epitope in Nef. J. Infect. Dis. *197*, 871–879.
38. Clayton, K.L., Collins, D.R., Lengieza, J., Ghebremichael, M., Dotiwala, F., Lieberman, J., and Walker, B.D. (2018). Resistance of HIV-infected macrophages to CD8+ T lymphocyte–mediated killing drives activation of the immune system. Nat. Immunol. *19*, 475–486.
39. Hellman, L.M., Yin, L., Wang, Y., Blevins, S.J., Riley, T.P., Belden, O.S., Spear, T.T., Nishimura, M.I., Stern, L.J., and Baker, B.M. (2016). Differential scanning fluorimetry based assessments of the thermal and kinetic stability of peptide-MHC complexes. J. Immunol. Methods *432*, 95–101.
40. Kaseke, C., Park, R.J., Singh, N.K., Koundakjian, D., Bashirova, A., Garcia Beltran, W.F., Takou Mbah, O.C., Ma, J., Senjobe, F., Urbach, J.M., et al. (2021). HLA class-I-peptide stability mediates CD8+ T cell immunodominance hierarchies and facilitates HLA-associated immune control of HIV. Cell Rep. *36*, 109378.
41. Stewart-Jones, G.B., Simpson, P., van der Merwe, P.A., Easterbrook, P., McMichael, A.J., Rowland-Jones, S.L., Jones, E.Y., and Gillespie, G.M. (2012). Structural features underlying T-cell receptor sensitivity to concealed MHC class I micropolymorphisms. Proc. Natl. Acad. Sci. USA *109*, E3483–E3492.
42. Blanco-Gelaz, M.A., Suárez-Alvarez, B., González, S., López-Vázquez, A., Martínez-Borra, J., and López-Larrea, C. (2006). The amino acid at position 97 is involved in folding and surface expression of HLA-B27. Int. Immunol. *18*, 211–220.
43. Pymm, P., Illing, P.T., Ramarathinam, S.H., O'Connor, G.M., Hughes, V.A., Hitchen, C., Price, D.A., Ho, B.K., McVicar, D.W., Brooks, A.G., et al. (2017). MHC-I peptides get out of the groove and enable a novel mechanism of HIV-1 escape. Nat. Struct. Mol. Biol. *24*, 387–394.
44. Kloverpris, H.N., Stryhn, A., Harndahl, M., van der Stok, M., Payne, R.P., Matthews, P.C., Chen, F., Riddell, L., Walker, B.D., Ndung'u, T., et al. (2012). HLA-B*57 Micropolymorphism shapes HLA allele-specific epitope immunogenicity, selection pressure, and HIV immune control. J. Virol. *86*, 919–929.
45. Migueles, S.A., Osborne, C.M., Royce, C., Compton, A.A., Joshi, R.P., Weeks, K.A., Rood, J.E., Berkley, A.M., Sacha, J.B., Cogliano-Shutta, N.A., et al. (2008). Lytic granule loading of CD8+ T cells is required for HIV-infected cell elimination associated with immune control. Immunity *29*, 1009–1021.
46. Gaiha, G.D., McKim, K.J., Woods, M., Pertel, T., Rohrbach, J., Barteneva, N., Chin, C.R., Liu, D., Soghoian, D.Z., Cesa, K., et al. (2014). Dysfunctional HIV-specific CD8+ T cell proliferation is associated with increased caspase-8 activity and mediated by necroptosis. Immunity *41*, 1001–1012.
47. Collins, D.R., Urbach, J.M., Racenet, Z.J., Arshad, U., Power, K.A., Newman, R.M., Mylvaganam, G.H., Ly, N.L., Lian, X., Rull, A., et al. (2021). Functional impairment of HIV-specific CD8+ T cells precedes aborted spontaneous control of viremia. Immunity *54*, 2372–2384.e7.
48. McLaren, P.J., Ripke, S., Pelak, K., Weintrob, A.C., Patsopoulos, N.A., Jia, X., Erlich, R.L., Lennon, N.J., Kadie, C.M., Heckerman, D., et al. (2012). Fine-mapping classical HLA variation associated with durable host control of HIV-1 infection in African Americans. Hum. Mol. Genet. *21*, 4334–4347.
49. Held, W., Dorfman, J.R., Wu, M.F., and Raulet, D.H. (1996). Major histocompatibility complex class I-dependent skewing of the natural killer cell Ly49 receptor repertoire. Eur. J. Immunol. *26*, 2286–2292.
50. Dorfman, J.R., and Raulet, D.H. (1998). Acquisition of Ly49 receptor expression by developing natural killer cells. J. Exp. Med. *187*, 609–618.
51. Khakoo, S.I., Thio, C.L., Martin, M.P., Brooks, C.R., Gao, X., Astemborski, J., Cheng, J., Goedert, J.J., Vlahov, D., Hilgartner, M., et al. (2004). HLA and NK cell inhibitory receptor genes in resolving hepatitis C virus infection. Science *305*, 872–874.
52. Parikh, B.A., Bern, M.D., Piersma, S.J., Yang, L., Beckman, D.L., Poursine-Laurent, J., Plougastel-Douglas, B., and Yokoyama, W.M. (2020). Control of Viral Infection by Natural Killer Cell Inhibitory Receptors. Cell Rep. *32*, 107969.
53. Anfossi, N., André, P., Guia, S., Falk, C.S., Roetynck, S., Stewart, C.A., Breso, V., Frassati, C., Reviron, D., Middleton, D., et al. (2006). Human NK cell education by inhibitory receptors for MHC class I. Immunity *25*, 331–342.
54. Gillespie, G.M.A., Stewart-Jones, G., Rengasamy, J., Beattie, T., Bwayo, J.J., Plummer, F.A., Kaul, R., McMichael, A.J., Easterbrook, P., Dong, T., et al. (2006). Strong TCR conservation and altered T cell cross-reactivity characterize a B*57-restricted immune response in HIV-1 infection. J. Immunol. *177*, 3893–3902.
55. Chatzileontiadou, D.S.M., Lobos, C.A., Robson, H., Almedia, C.-A., Szeto, C., D'Orsogna, L.J., and Gras, S. (2023). Public T Cell Clonotypes Are Selected in HLA-B*57:01/HIV Patients Independently of the Viral Load. https://doi.org/10.2139/ssrn.4439674.
56. Mendoza, D., Royce, C., Ruff, L.E., Ambrozak, D.R., Quigley, M.F., Dang, T., Venturi, V., Price, D.A., Douek, D.C., Migueles, S.A., et al. (2012). HLA B*5701-positive long-term nonprogressors/elite controllers are not distinguished from progressors by the clonal composition of HIV-specific CD8+ T cells. J. Virol. *86*, 4014–4018.
57. Koning, D., Quakkelaar, E.D., Schellens, I.M.M., Spierings, E., and van Baarle, D. (2022). Protective HLA Alleles Recruit Biased and Largely Similar Antigen-Specific T Cell Repertoires across Different Outcomes in HIV Infection. J. Immunol. *208*, 3–15.
58. Chapman, T.L., Heikeman, A.P., and Bjorkman, P.J. (1999). The inhibitory receptor LIR-1 uses a common binding interaction to recognize class I MHC molecules and the viral homolog UL18. Immunity *11*, 603–613.
59. Jones, D.C., Kosmoliaptsis, V., Apps, R., Lapaque, N., Smith, I., Kono, A., Chang, C., Boyle, L.H., Taylor, C.J., Trowsdale, J., et al. (2011). HLA class I allelic sequence and conformation regulate leukocyte Ig-like receptor binding. J. Immunol. *186*, 2990–2997.
60. Coleman, J.E., Huentelman, M.J., Kasparov, S., Metcalfe, B.L., Paton, J.F.R., Katovich, M.J., Semple-Rowland, S.L., and Raizada, M.K. (2003). Efficient large-scale production and concentration of HIV-1-based lentiviral vectors for use *in vivo*. Physiol. Genom. *12*, 221–228.
61. Pierce, B.G., Hellman, L.M., Hossain, M., Singh, N.K., Vander Kooi, C.W., Weng, Z., and Baker, B.M. (2014). Computational design of the affinity and specificity of a therapeutic T cell receptor. PLoS Comput. Biol. *10*, e1003478.

62. Shimizu, Y., and DeMars, R. (1989). Production of human cells expressing individual transferred HLA-A,-B,-C genes using an HLA-A,-B,-C null human cell line. J. Immunol. *142*, 3320–3328.

63. Walker, B.D., Flexner, C., Birch-Limberger, K., Fisher, L., Paradis, T.J., Aldovini, A., Young, R., Moss, B., and Schooley, R.T. (1989). Long-term culture and fine specificity of human cytotoxic T-lymphocyte clones reactive with human immunodeficiency virus type 1. Proc. Natl. Acad. Sci. USA *86*, 9514–9518.

64. Naranbhai, V., Nathan, A., Kaseke, C., Berrios, C., Khatri, A., Choi, S., Getz, M.A., Tano-Menka, R., Ofoman, O., Gayton, A., et al. (2022). T cell reactivity to the SARS-CoV-2 Omicron variant is preserved in most but not all individuals. Cell *185*, 1041–1051.e6.

65. Behrendt, R., White, P., and Offer, J. (2016). Advances in Fmoc solid-phase peptide synthesis. J. Pept. Sci. *22*, 4–27.

66. Chatzi, K.B.O., Gatos, D., and Stavropoulos, G. (1991). 2-Chlorotrityl chloride resin: Studies on anchoring of Fmoc-amino acids and peptide cleavage. Int. J. Pept. Protein Res. *37*, 513–520.

67. Teixeira, A., Benckhuijsen, W.E., de Koning, P.E., Valentijn, A.R.P.M., and Drijfhout, J.W. (2002). The use of DODT as a non-malodorous scavenger in Fmoc-based peptide synthesis. Protein Pept. Lett. *9*, 379–385.

68. Bethune, M.T., Comin-Anduix, B., Fu, Y.-H.H., Ribas, A., and Baltimore, D. (2017). Preparation of peptide–MHC and T-cell receptor dextramers by biotinylated dextran doping. Biotechniques *62*, 123–130.

## STAR★METHODS

### KEY RESOURCES TABLE

| REAGENT or RESOURCE | SOURCE | IDENTIFIER |
|---|---|---|
| Antibodies | | |
| Mouse monoclonal anti-HLA ABC (clone W6/32) labeled with APC fluorophore | Biolegend | Cat# 311410; RRID: AB_314878 |
| Mouse monoclonal anti-CD3 (clone UCHT1) labeled with PE/Cy7 fluorophore | Biolegend | Cat# 300420; RRID: AB_439781 |
| Mouse monoclonal anti-CD3 (clone UCHT1) labeled with PE fluorophore | Biolegend | Cat# 300408; RRID: AB_2564150 |
| Mouse monoclonal anti-CD69 (clone FN50) labeled with FITC fluorophore | Biolegend | Cat# 310904; RRID: AB_314839 |
| Mouse monoclonal anti-KIR3DL1 (clone DX9) labeled with APC fluorophore | Biolegend | Cat# 312716; RRID: AB_2563359 |
| Anti-CD3 antibody 12F6 | A gift from J. Wong | 12F6 |
| LIVE/DEAD Violet Viability | Thermo Fisher | Cat# L34960 |
| CellTrace FarRed Dye | Thermo Fisher | Cat# C34564 |
| Bacterial and virus strains | | |
| Stellar competent cells (*E.coli* HST08) | Takara Bio | Cat# 636763 |
| Biological samples | | |
| B57-restricted HIV-specific $CD8^+$ T cell clones | A gift from Bruce Walker, Ragon Institute | N/A |
| Polyclonal B57-restricted HIV-specific $CD8^+$ T cells | Ragon Institute $HIV^+$ Sample Repository | N/A |
| Chemicals, peptides, and recombinant proteins | | |
| B*5701 HIV epitope peptides | MGH Peptide Core | N/A |
| Soluble HLA-B*5701 HIV peptide monomers | This paper | N/A |
| HLA-B*5701 HIV peptide dextramers | This paper | N/A |
| KIR3DL1-Fc | RnD Systems | Cat# 1225-KR |
| Critical commercial assays | | |
| Human IFN-γ ELISpot Assay (Flex) | Mabtech | Cat# 3420-2A |
| QiaPrep Miniprep Spin Kit | Qiagen | Cat# 27104 |
| Nucleobond Xtra Maxi EF | Macherey-Nagel | Cat# 740424.50 |
| Experimental models: Cell lines | | |
| Human: 721.221 cells | A gift from Bruce Walker, Ragon Institute | N/A |
| Human: HEK293T cells | ATCC | CRK-1573 |
| Human: 721.221 cells + HLA-B*5701 wildtype and mutants | This paper | N/A |
| Human: Jurkat-KIR3DL1-CD3ζ reporter cells | A gift from Wilfredo Garcia Beltran | N/A |
| Oligonucleotides | | |
| Primers for site-directed mutagenesis to generate HLA-B*5701 mutants (Table S2) | This paper | N/A |
| Recombinant DNA | | |
| psPAX2 | A gift of Didier Trono, EPFL | Addgene Plasmid #12260; RRID: Addgene_12260 |
| pHEF-VSVG | [60] | Addgene Plasmid #22501; RRID: Addgene_22501 |
| pLVX-EF1α-IRES-Puro | Clontech | Cat# 631988; RRID: Addgene_85132 |

(*Continued on next page*)

| Continued | | |
|---|---|---|
| REAGENT or RESOURCE | SOURCE | IDENTIFIER |
| pLVX-SFFV-IRES-Puro | This paper | N/A |
| pLVX-SFFV-IRES-Puro + HLA-B*57 + ZsGreen Inserts | This paper | N/A |
| pET28a with HLA-B*5701 wild-type and mutant and β2M | [61] | N/A |
| Software and algorithms | | |
| Prism 9 | GraphPad Software | https://www.graphpad.com/scientific-software/prism/ |
| FlowJo | BD | https://www.flowjo.com |

## RESOURCE AVAILABILITY

### Lead contact

Further information and requests for resources and reagents should be directed to and will be fulfilled by the lead contact, Gaurav D. Gaiha (ggaiha@mgh.harvard.edu).

### Materials availability

All requests for resources and reagents should be directed to and will be fulfilled by the lead contact author. All reagents will be made available on request after completion of a Materials Transfer Agreement.

### Data and code availability

- All data reported in this paper will be shared by the lead contact upon request.
- This paper does not report original code.
- Any additional information required to reanalyze the data reported in this paper is available from the lead contact upon request.

## EXPERIMENTAL MODEL AND STUDY PARTICIPANT DETAILS

### Cell lines

The human female B cell line 721.221 were generated previously by γ-radiation of 721 cells and do not express HLA A and B alleles.[62] These cell lines were maintained in RPMI-1640 medium (Sigma-Aldrich) supplemented with 10% (v/v) FBS (Sigma-Aldrich) and 1X Penicillin-Streptomycin-L-Glutamine mixture (Gibco). HEK293T cells used for lentivirus production were maintained in advanced DMEM (Sigma-Aldrich) supplemented with 10% FBS, 2mM L-glutamine (Gibco), 1X non-essential amino acids (Gibco) and 1X sodium pyruvate (Gibco). Jurkat cells stably transduced with a chimeric KIR3DL1 receptor linked to the cytoplasmic tail of CD3ζ as reported previously[34] were maintained in RPMI-1640 medium (Sigma-Aldrich) supplemented with 10% (v/v) FBS (Sigma-Aldrich) and 1X Penicillin-Streptomycin-L-Glutamine mixture (Gibco).

### HLA-B*5701 wild-type and mutant-expressing cell line generation

HEK293T cells were transfected with psPAX2, pHEF-VSVG and pLVX-SFFV-ZsGreen-P2A-HLA-B*5701-IRES-Puro vector in a 3:1:4 ratio using Lipofectamine 3000 (Thermo Fisher Scientific) in OptiMem media (Gibco). Media was changed to Dulbecco's Modified Eagle's Medium (Sigma-Aldrich) supplemented with 10% (v/v) FBS (Sigma-Aldrich) 24h post-transfection. After 48h, lentivirus was harvested by filtering supernatant through a 0.45 μm low protein binding durapore membrane (Millipore). Frozen aliquots were stored at -80°C. 721.221 cells were subsequently transduced with lentivirus encoding wild-type or mutant HLA-B*5701 genes and selected in 0.5 μg/ml puromycin (Invivogen). Surface HLA-B*5701 expression was confirmed by staining with pan-HLA class I antibody W6/32 (1:100; Biolegend),[36] fixation in 4% paraformaldehyde and flow cytometric analysis using a BD LSR II (BD Biosciences). Flow cytometric data were analyzed using FlowJo software (v10.1r5; Treestar).

### HIV-specific CD8$^+$ T cell clone generation

Peripheral blood mononuclear cells (PBMCs) were stimulated with cognate HIV peptide for 90 minutes prior to culturing for 14 days in RPMI-1640 medium (Sigma-Aldrich) supplemented with 10% (v/v) FBS (Sigma-Aldrich) and 50U/mL of recombinant IL-2 (Peprotech). Expansion of epitope-specific CD8$^+$ T cells was confirmed by IFN-γ ELISpot prior to limited dilution of single cells into individual wells of 96 well U-bottom plates. Clones were stimulated with irradiated allogeneic PBMCs and monoclonal anti-CD3 antibody12F6 (a gift from J. Wong) as a stimulus for T cell proliferation.[63] Developing epitope-specific CD8$^+$ T clones were confirmed by IFN-γ ELISpot with optimal epitopes and tetramer staining. Cloned CD8$^+$ T cells were subsequently maintained by restimulation every 14-21 days with 12F6 antibody and irradiated allogeneic PBMCs in RPMI-1640 medium containing 50 U/ml of recombinant IL-2, as described.[63]

### Study participants

HIV+ B*5701+ study participants were recruited from outpatient clinics at local Boston area clinics and from outside Boston. The Institutional Review Board of Massachusetts General Hospital approved the studies of cells derived from human blood samples. All human subjects gave written, informed consent. Peripheral blood mononuclear cells (PBMCs) from HIV+ individuals were collected by Ficoll gradient separation from ACD tubes or leukapheresis samples. They were then cryopreserved and stored in liquid nitrogen for future use. High resolution HLA class I-typing was performed for all patients as described previously.[64] Briefly, locus-specific PCR primers were used to amplify polymorphic exons of *HLA-A, HLA-B, HLA-C* genes with the Fluidigm Access Array (Fluidigm). PCR amplicons were pooled and sequenced on an Illumina MiSeq platform (Illumina). *HLA* alleles and genotypes were called using the Omixon HLA Explore (beta version) software (Omixon). Ambiguous calls were resolved by Sanger sequencing.

## METHOD DETAILS

### Recombinant DNA constructs

The plasmid psPAX2 was a gift from Didier Trono (Addgene plasmid # 12260; http://n2t.net/addgene:12260; RRID:Addgene_12260). The plasmid pHEF-VSVG was a gift from Sergey Kasparov (Addgene plasmid # 22501; http://n2t.net/addgene:22501; RRID:Addgene_22501). The synthetic HLA-B*5701 allele fragment (LifeSct) was cloned into a modified pLVX-EF1α-IRES-Puro vector (Clontech), in which EF1α was replaced with the SFFV promoter (pLVX-SFFV-IRES-Puro). This expression cassette also encoded ZsGreen linked via self-cleaved P2A peptide to HLA with a FLAG-tag at its N-terminus. The pET28a vector (Takara Bio) was used for all soluble protein expression in *E.coli*. All plasmids were confirmed by complete plasmid sequencing (MGH DNA Core).

### Site-directed mutagenesis

Individual mutations to HLA-B*5701 were introduced using the Q5 Site-Directed Mutagenesis Kit (New England Biolabs) according to the manufacturer's instructions using back-to-back 5' oligonucleotide primers (Table S2) within the pLVX-SFFV-ZsGreen-P2A-HLA-B*5701-IRES-Puro vector. Confirmation of successful mutagenesis was accomplished by complete plasmid sequencing (MGH Sequencing Core). Full-length plasmids were propagated in Stellar competent cells (Takara Bio) and DNA plasmid stocks were prepared using a QiaPrep spin miniprep kit (Qiagen). The L156R mutation was engineered by synthesizing a mutant HLA-B*5701 DNA fragment (Integrated DNA Technologies) and cloning into the lentiviral expression vector.

### Isolation of primary CD8+ T cells from HIV+ individuals

Isolation of CD8+ T cells from HIV+ individuals was performed using magnetic anti-CD8+ beads and the MACS cell separation system (Miltenyi Biotec) according to the manufacturer's instructions. All cell enrichment procedures were conducted by positive selection.

### Peptide synthesis reagents

Fmoc-protected amino acids and synthesis resin, 2-Chlorotrityl chloride were purchased from Akaal Organics (Long Beach, CA). Dimethylformamide (DMF), N-methyl pyrrolidone (NMP), Acetonitrile and Methyl-tert. Butyl Ether (MTBE) were purchased from Fisher Bioreagents (Fair Lawn, NJ). 2-(6-Chloro-1-H-benzotriazole-1-yl)-1,1,3,3-tetramethylaminium hexafluorophosphate (HCTU) was purchased from AAPPTEC (Louisville, KY). Piperidine and Dichloromethane (DCM) were from EMD-Millipore (Billerica, MA). Diisopropylethylamine (DIEA), N-Methyl-morpholine (NMM), Triisoprpopyl-silane, 3,6-dioxa-1,8-octanedithiol (DODT) and trifluoroacetic acid (TFA) were purchased from Sigma–Aldrich.

### Peptide synthesis and analysis

Peptides were synthesized on an automated robotic peptide synthesizer (AAPPTEC, Model 396 Omega) by using Fmoc solid-phase chemistry[65] on 2-chlorotrityl chloride resin.[66] The C-terminal amino acids were loaded using the respective Fmoc-Amino Acids in the presence of DIEA. Unreacted sites on the resin were blocked using methanol, DIEA and DCM (15:5:80 v/v). Subsequent amino acids were coupled using optimized (to generate peptides containing more than 90% of the desired full-length peptides) cycles consisting of Fmoc removal (deprotection) with 25% Piperidine in NMP followed by coupling of Fmoc-AAs using HCTU/NMM activation. Each deprotection or coupling was followed by several washes of the resin with DMF to remove excess reagents. After the peptides were assembled and the final Fmoc group removed, peptide resin was then washed with dimethylformamide, dichloromethane, and methanol three times each and air dried. Peptides were cleaved from the solid support and deprotected using odor free cocktail (TFA/triisopropyl silane/water/DODT; 94/2.5/2.5/1.0 v/v) for 2.5h at room temperature.[67] Peptides were precipitated using cold methyl tertiary butyl ether (MTBE). The precipitate was washed 2 times in MTBE, dissolved in a solvent (0.1% trifluoroacetic acid in 30%Acetonitrile/70%water) followed by freeze drying. Peptides were characterized by Ultra Performance Liquid Chromatography (UPLC) and Matrix Assisted Laser Desorption/Ionization Mass Spectrometry (MALDI-MS). All peptides were dissolved initially in 100% DMSO at a concentration of 40 mM, prior to dilution at the appropriate concentration in RPMI-1640 medium.

### CD8+ T cell recognition assay

Wild-type and mutant HLA-B*5701-expressing 721.221 target cells were pulsed for 1hr at 37°C with 1μM of HIV peptide, washed and co-cultured with either HIV-specific CD8+ T cell clones (at an effector:target ratio of 1:10) or purified CD8+ T cells from

B*5701+ HIV-infected individuals (at an effector:target ratio of 1:1) at 50,000 target cell/well in a 96-well nitrocellulose plate (Millipore) coated with anti-human IFN-γ antibody. Co-cultures were incubated for 16-18 hours prior to assessment of CD8+ T cell reactivity by IFN-γ ELISpot assay performed according to the manufacturer's instructions (Mabtech).

### CD8+ T cell elimination assay

Elimination assays were performed as previously described[38] with modifications. 50% of wild-type and mutant HLA-B*5701-expressing 721.221 target cells were pulsed for 1hr at 37°C with 1μM of HIV peptide, washed and labeled with CellTrace Far Red dye (Thermo Fisher) prior to mixing with unpulsed target cells in a 1:1 ratio. Targets cells were then co-cultured with HIV-specific CD8+ T cell clones at an effector:target (E:T) ratio of 1:1 with 50,000 target cells/well in a treated 96-well polystyrene plate (Corning) for 16-18h. Co-cultures were then stained with anti-CD3-PECy7 antibody (Biolegend) and viability dye (violet; Thermo Fisher) and elimination was determined by the percentage of residual peptide-pulsed cells (scatter-intact, singlet, Live/Dead violet-, CD3-, Far Red+) by flow cytometry (BD LSR II, BD Biosciences). %Elimination was determined by the following formula: (1 - (%FarRed+ with CD8+ T cell clone/%FarRed+ without CD8+ T cell clone).

### Soluble HLA class I-peptide monomer expression and purification

Expression, refold and purification of the soluble constructs of HLA class I-peptide monomer were performed as previously described.[61] Briefly, HLA-B*5701 heavy chains and β2-microglobulin were expressed in *E. coli* and purified as inclusion bodies (IBs). The IBs were solubilized in 8M Urea. Target peptides were provided by the MGH Peptide Core. For the refold, HLA-B*5701 wild-type or mutant heavy chain, β2m and the peptide (~1mM final concentration) were added to a refolding buffer containing 100mM Tris at pH 8.3, 2mM EDTA, 400mM L-arginine, 4M Urea, 1 mM oxidized glutathione, 1.5 mM reduced glutathione, and 0.2mM PMSF in a molar ratio of 1:3:5 respectively. The reaction mix was first incubated at 4°C for 24 hours and followed by dialysis against 10 mM Tris for the next 60 hours at 4°C. The HLA class I-peptide complexes were purified using DEAE column followed by size exclusion chromatography.

### Thermal denaturation assay

Thermal denaturation was performed by differential scanning fluorimetry using a Bio-RAD CFX96 real time PCR system as previously described.[40] Briefly, the excitation and emission wavelengths were set to 587 and 607 nm respectively, and the fluorescence intensity was measured after every 1°C rise in temperature starting from 20°C and going up to 95°C. Each reaction mix contained 19.8μL of 2μM HLA class I-peptide (buffer: 10mM HEPES at pH 7.4, 150 mM NaCl, 3 mM EDTA, and 0.005% surfactant P20) and 0.2μL of 1000X SYPRO orange dye. Apparent $T_m$ values were calculated by identifying the point at which the melting transition was 50% complete.

### Structural analysis of HLA-B*5701-peptide complexes

All mutations were analyzed in the HLA-B*5701 structure in complexed with TW10 (PDB: 5V5M) or the closely related HLA-B*5703 structure in complex with KF11 (PDB: 2YPK). The HLA-B*3501 structure (PDB: 3LKO) having F67 and N70 was used for the M67F and S70N models within HLA-B*57 based on structure alignment. Similarly, the HLA-B*0702 structure (PDB: 5EO0) used for Y67, Q70 and R156 model building in HLA-B*5701, HLA-B*5703 and HLA-B*2705 structure (PDB: 4G9D) was used for K70. For the analysis of M67A, M67S and S70A mutations, we used the Mutagenesis and Backbone Rotamers function in Pymol to build the models. All figures were drawn and polished by the PyMOL Molecular Graphics System (Version 2.0 Schrödinger, LLC).

### Generation of fluorescently labeled HLA-B*5701-peptide tetramers

Soluble HLA-B*5701 wild-type and mutant monomers were biotinylated using BirA ligase in presence of 100-fold excess biotin. Post biotinylation, soluble HLA class I-peptide monomers were purified on a S200 size exclusion column. Fluorescently labeled tetramers were then produced by multimerization with PE-conjugated streptavidin (Biolegend) per the manufacturer's instructions. Binding of PE-labeled tetramers to HIV-specific CD8+ T cell clones was determined by incubation at 37°C for 30 min prior to staining with monoclonal antibodies (anti-CD3 PE-Cy7, anti-CD8 APC; Biolegend) and viability dye (violet; Thermo Fisher) and analysis by flow cytometry using a BD LSR II (BD Biosciences).

### KIR3DL1 surface plasmon resonance

KIR3DL1 Binding measurements were performed using surface plasmon resonance (SPR) on a Biacore T200 instrument. KIR3DL1-Fc (R&D Systems) was coupled to a series S Protein A chip (Biacore) at a density of ~1000 RU. Soluble monomers of HLA-B*5701-TW10 and HLA-B*5701 V97S-TW10 were injected over ligand coated chip at concentration series ranging from 0μM to 193μM and 0μM to 234μM respectively. Each injection contact time was 300s followed by a dissociation time of 700s. The experiment was performed at 25°C, and all the proteins were maintained in HBS-EP+ buffer. The data was analyzed on a BiaEval software, assuming 1:1 binding model, and plotted using Origin software.

### Dextramer assembly

HLA-B*5701, HLA-B*5701 V97S mutant and β2-microglobulin were expressed in E. coli, refolded in the presence of TW10 peptide, and biotinylated using BirA ligase in presence of 100-fold excess biotin. Post biotinylation, soluble HLA class I-peptide monomers

were purified on a S200 size exclusion column and HLA class I-peptide dextramers were prepared as described previously.[68] Briefly, soluble HLA class I-peptide trimer were prepared by adding one equivalent (eq) (2.5μM final) of fluorescently labeled APC-streptavidin over 10 × 10 min time intervals to 3eq biotin-HLA class I-peptide monomers (7.5μM final). Biotinylated dextran (Molecular weight 500kDa; Molecular Probes) was then added to the trimer mix at 1:20 with respect to streptavidin (125nM final) and incubated for 10 min prior to immediate use in staining experiments.

### KIR3DL1 reporter cell assay

In the Jurkat-KIR3DL1-CD3ζ cells (a gift from Wilfredo Garcia-Beltran, Ragon Institute), ligand engagement of surface KIR3DL1 results in an activating signal that triggers CD69 expression, making them suitable as a cell reporter system as previously described.[34] Prior to co-incubation experiments, Jurkat-KIR3DL1-CD3ζ cells were rested for 24h in 20% (v/v) FBS (Sigma-Aldrich). 721.221-B*5701 cells (wild-type or mutant) were pulsed for 1hr at 37°C with 1μM of HIV peptide, washed and then co-cultured with rested Jurkat-KIR3DL1-CD3ζ cells for 16-18h. Co-cultures were then stained with viability dye, anti-CD3-PE, anti-KIR3DL1-APC and anti-CD69-FITC antibodies prior to analysis by flow cytometry (BD LSR II, BD Biosciences). Jurkat-KIR3DL1-CD3ζ cells were identified by gating on KIR3DL1$^{hi}$CD3$^{+}$ cells in the viable cell population. KIR3DL1 binding was determined by percentage of KIR3DL1$^{hi}$CD3$^{+}$ that expressed CD69 relative to background controls.

## QUANTIFICATION AND STATISTICAL ANALYSIS

The generation of dot plots, nonparametric statistical analysis and correction for multiple comparisons were performed using the statistical programs in Graphpad Prism version 8.4.1. Differences between groups were evaluated using unpaired *t*-test. All statistical details and p-values can be found in figure legends.

**Structure, Volume *32***

## Supplemental Information

# Polymorphic residues in *HLA-B* that mediate HIV control distinctly modulate peptide interactions with both TCR and KIR molecules

**Rhoda Tano-Menka, Nishant K. Singh, Itai Muzhingi, Xiaolong Li, Michael V. Mandanas, Clarety Kaseke, Charles R. Crain, Angela Zhang, Funsho J. Ogunshola, Liza Vecchiarello, Alicja Piechocka-Trocha, Arman Bashirova, Michael E. Birnbaum, Mary Carrington, Bruce D. Walker, and Gaurav D. Gaiha**

| HLA-B*5701 AA Mutation(s) | Notable HLA Alleles with Mutant Amino Acids |
|---|---|
| M67Y | B*0702 |
| M67F | B*0801, B*3501 |
| M67S | B*5201, Cw*07 |
| M67A | Ala Mutant |
| S70Q | B*0702, B*0801 |
| S70N | B*0801, B*1402, B*3501, Cw*0701 |
| S70K | B*2705 |
| S70A | Ala Mutant |
| V97S | B*0702, B*0801 |
| V97R | B*3501, B*5801 |
| V97T | B*5201 |
| V97A | Ala Mutant |
| M67YS70Q | B*0702 |
| M67YV97S | B*0702 |
| S70QV97S | B*0702 |
| M67YS70QV97S | B*0702 |
| L156R | B*0702 |

**Table S1. Mutations engineered in HLA-B*5701, related to Figures 2-8.** The table depicts the engineered mutations in the HLA-B*5701 lentiviral expression plasmid and the protective and risk alleles in which these amino acid residues are naturally found. Each amino acid was also mutated to alanine.

| AA Mutation | Forward Primer (Mutant) | Reverse Primer | Ta (∘C) |
|---|---|---|---|
| M67Y | GACACGGAACTATAAGGCCTCCGCGCAG | TCCCCGTCCCAATACTCC | 63 |
| M67F | GACACGGAACTTTAAGGCCTCCG | TCCCCGTCCCAATACTCC | 65 |
| M67S | GACACGGAACTCTAAGGCCTCCGC | TCCCCGTCCCAATACTCC | 67 |
| M67A | GACACGGAACGCAAAGGCCTCCGC | TCCCCGTCCCAATACTCC | 63 |
| S70Q | CATGAAGGCCCAGGCGCAGACTTACCG | TTCCGTGTCTCCCCGTCC | 63 |
| S70N | CATGAAGGCCAATGCGCAGACTTACCGAG | TTCCGTGTCTCCCCGTCC | 71 |
| S70K | CATGAAGGCCAAAGCGCAGACTTACCGAG | TTCCGTGTCTCCCCGTCC | 67 |
| S70A | CATGAAGGCCGCTGCGCAGACTTAC | TTCCGTGTCTCCCCGTCC | 69 |
| V97S | CATCATCCAGCGAATGTATGGCTGCGACGTGG | TGAGACCCGGCCTCGCTC | 60 |
| V97R | CATCATCCAGTCTATGTATGGCTGCGACGTGGG | TGAGACCCGGCCTCGCTC | 60 |
| V97T | CATCATCCAGACTATGTATGGCTGCGACGTGG | TGAGACCCGGCCTCGCTC | 60 |
| V97A | CATCATCCAGGCAATGTATGGCTGCGACG | TGAGACCCGGCCTCGCTC | 60 |
| M67YS70Q | GACACGGAACTATAAGGCCTCCGCGCAG | TCCCCGTCCCAATACTCC | 63 |
| M67YV97S | CATCATCCAGCGAATGTATGGCTGCGACGTGG | TTCCGTGTCTCCCCGTCC | 60 |
| S70QV97S | CATCATCCAGCGAATGTATGGCTGCGACGTGG | TTCCGTGTCTCCCCGTCC | 60 |
| M67YS70QV97S | CATCATCCAGCGAATGTATGGCTGCGACGTGG | TTCCGTGTCTCCCCGTCC | 60 |

**Table S2. Primers utilized to engineer mutations in HLA-B*5701, related to Figures 2-8.** The table depicts the forward and reverse primers utilized to engineer mutations in the HLA-B*5701 lentiviral expression plasmid. Mutant codons highlighted in red text. Of note, the L156R mutation was engineered by synthesizing a mutant HLA-B*5701 DNA fragment and cloning into either the lentiviral expression vector or soluble HLA-B*5701 bacterial expression vector.

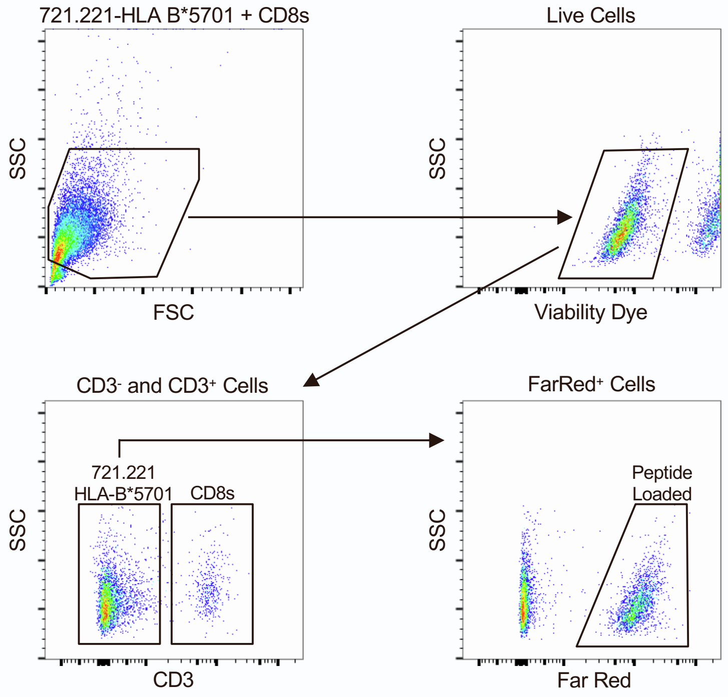


**Figure S1. Flow cytometry gating strategy for CD8+ T cell elimination assay, related to Figures 3 and 8.** HIV-specific CD8+ T cell clones that target an HLA-B*5701 epitope and 721.221-B*5701 wild-type and mutant cell lines were stained with viability dye and anti-CD3-PE antibody. 721.221-B*5701 cells were gated by absence of CD3 expression in the viable cell population. Peptide-loaded 721.221-B*5701 wild-type and mutant cells were subsequently gated by positive FarRed staining. The percentage decrease of the peptide-loaded FarRed population was used to determine the specific elimination of target 721-221-B*5701 cells by CD8+ T cell clones.

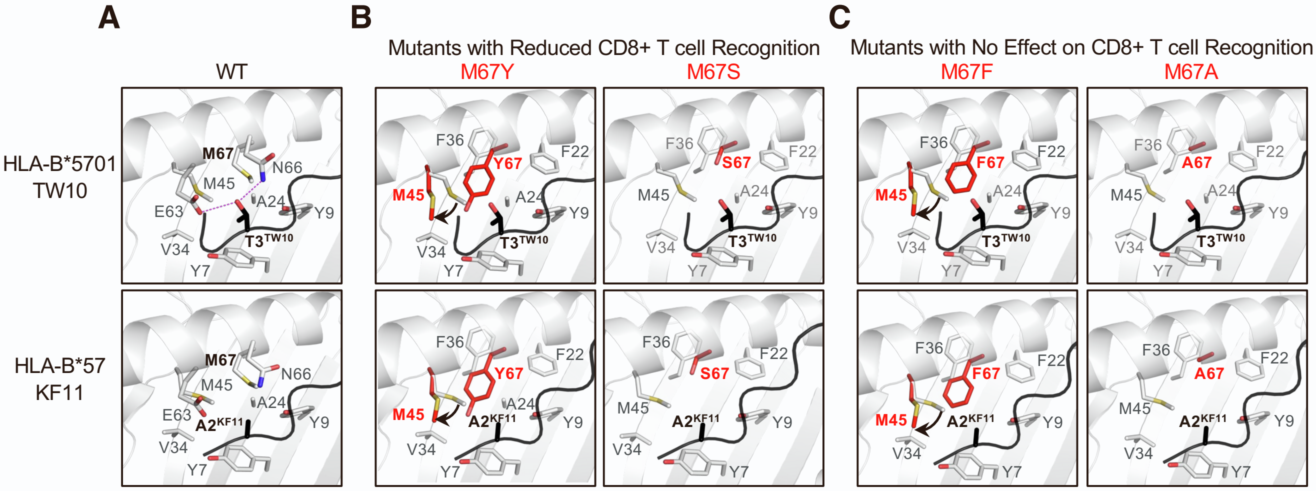


**Figure S2. Structural analysis of HLA-B*57 wildtype and M67 mutants in complex with the TW10 and KF11 peptide, related to Figure 5. (A)** Structural analysis of wild-type HLA-B*5701 presenting TW10 (PDB: 5V5M) and the closely related HLA-B*5703 presenting KF11 (PDB: 2YPK). HLA-B*57 was shown as grey ribbon and stick, while peptides were shown in black ribbon and stick. **(B)** Structural analysis of mutations that significantly affected CD8$^+$ T cell recognition and elimination (M67Y, M67S; **Figures 1-3**). The HLA-B*0702 structure (PDB: 5EO0) used to model Y67 and the Mutagenesis and Backbone Rotamers function in Pymol was used to model M67S. **(C)** Structural analysis of mutations that had no effect of CD8$^+$ T cell recognition and elimination (M67F, M67A; **Figures 1-3**). The HLA-B*3501 structure (PDB: 3LKO) was used to model M67F and the Mutagenesis and Backbone Rotamers function in Pymol was used to model M67A.

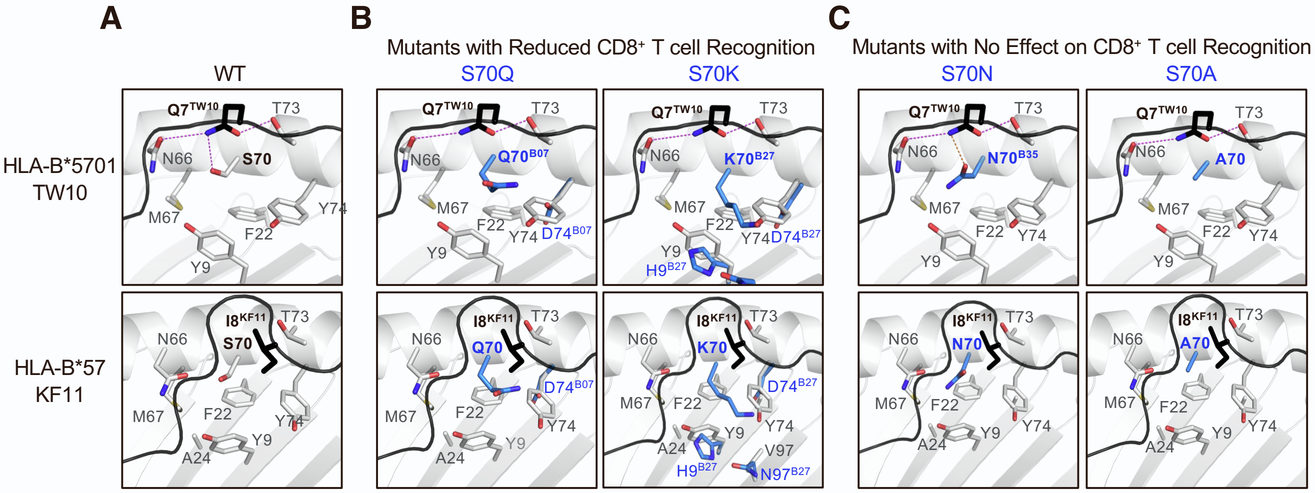


**Figure S3. Structural analysis of HLA-B*57 wildtype and S70 mutants in complex with the TW10 and KF11 peptide, related to Figure 6. (A)** Structural analysis of wild-type HLA-B*5701 presenting TW10 (PDB: 5V5M) and the closely related HLA-B*5703 presenting KF11 (PDB: 2YPK). HLA-B*57 was shown as grey ribbon and stick, while peptides were shown in black ribbon and stick. **(B)** Structural analysis of mutations that significantly affected CD8+ T cell recognition and elimination (S70Q, S70K; **Figures 1-3**). The HLA-B*0702 structure (PDB: 5EO0) used to model Q70 and the HLA-B*2705 structure (PDB: 4G9D) was used to model K70. **(C)** Structural analysis of mutations that had no effect of CD8+ T cell recognition and elimination (S70N, S70A; **Figures 1-3**). The HLA-B*3501 structure (PDB: 3LKO) was used to model S70N and the Mutagenesis and Backbone Rotamers function in Pymol was used to model S70A.

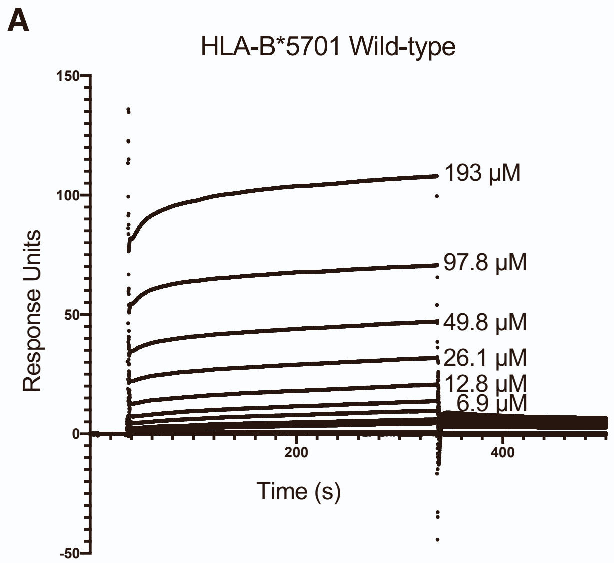


**Figure S4. Representative surface plasmon resonance (SPR) of soluble HLA-B*5701 wild-type and mutant V97S monomers to immobilized KIR3DL1-Fc, related to Figure 7. (A)** Representative surface plasmon resonance (SPR) injection series for soluble HLA-B*5701-TW10 complexes binding to immobilized KIR3DL1-Fc KIR3DL1 at range of concentrations, with a peak concentration of 193µM. **(B)** Representative surface plasmon resonance (SPR) injection series for soluble HLA-B*5701 V97S mutant-TW10 complexes binding to immobilized KIR3DL1-Fc at a range of concentrations, with a peak concentration of 234µM.

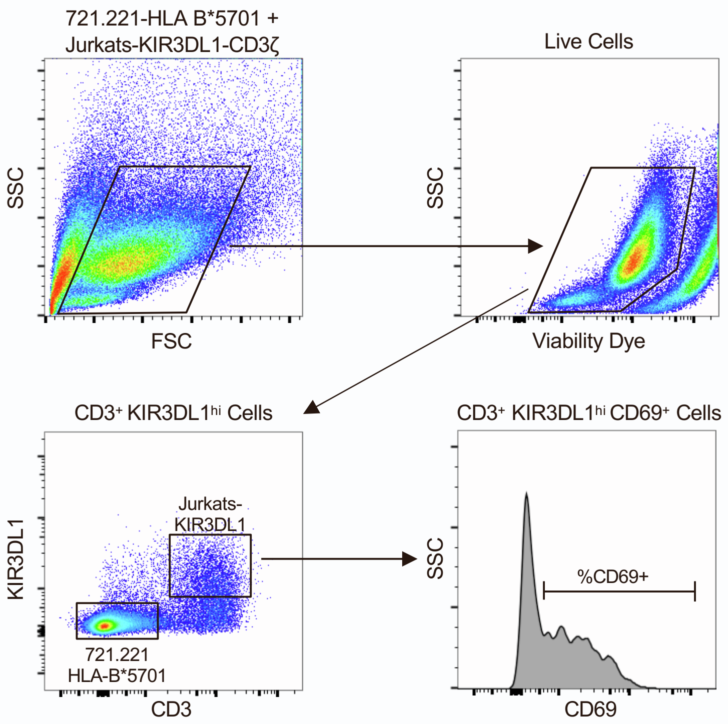


**Figure S5. Flow cytometry gating strategy for Jurkat-KIR3DL1-CD3ζ reporter cell assay, related to Figures 7 and 8.** Jurkat-KIR3DL1-CD3ζ cells and 721.221-B*5701 cell lines (wild-type and mutant) were stained with viability dye, anti-CD3-PE, anti-KIR3DL1-APC and anti-CD69-FITC antibodies. Jurkat-KIR3DL1-CD3ζ cells were identified by gating on KIR3DL1$^{hi}$CD3$^{+}$ cells in the viable cell population. Binding to KIR3DL1 was determined by percentage of KIR3DL1$^{hi}$CD3$^{+}$ that expressed CD69.

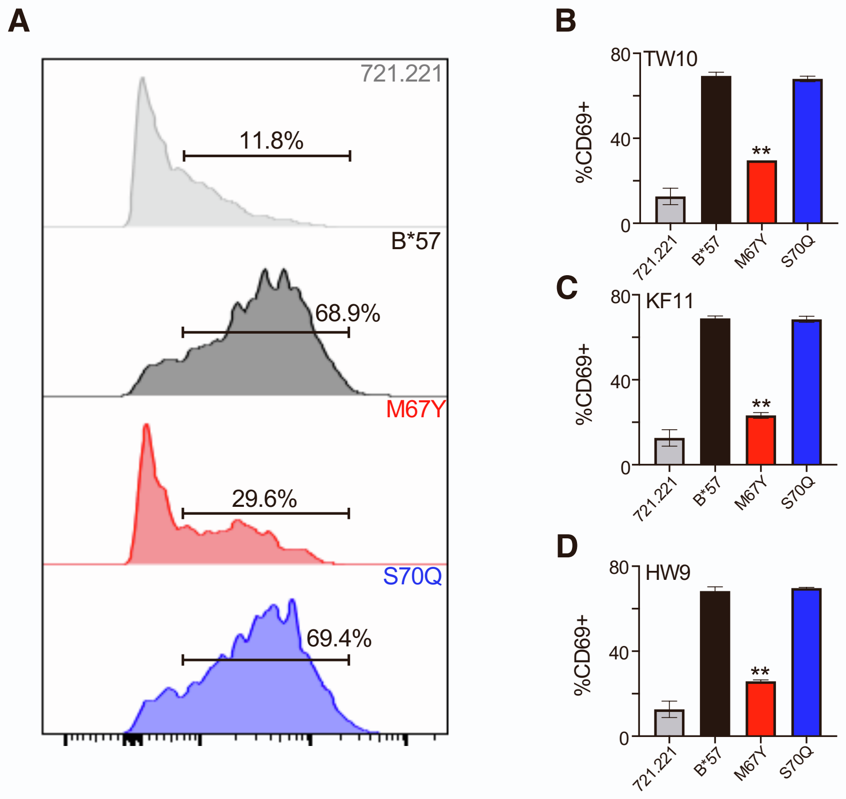


**Figure S6. Effect of B*5701 M67 and S70 mutations on KIR3DL1 interactions, related to Figure 7. (A)** Representative flow histograms of CD69 expression on $CD3^+$ $KIR3DL1^+$ Jurkat cells following co-culture with HLA-null 721.221 cells (gray), HLA-B*5701 cells (black), mutant M67Y (red) or mutant S70Q (blue) cells pulsed with TW10 peptide. (B-D) Comparison of $\%CD69^+$ $CD3^+$ $KIR3DL1^+$ Jurkat cells following co-culture with 721.221, wild-type HLA-B*5701, M67Y or S70Q mutant HLA-B*5701-expressing cell lines pulsed with TW10 peptide, KF11 peptide or HW9 peptide respectively. Error bars for (B-D) indicate standard deviation from three biological replicates performed independently. Statistical comparisons were made using an unpaired t-test.

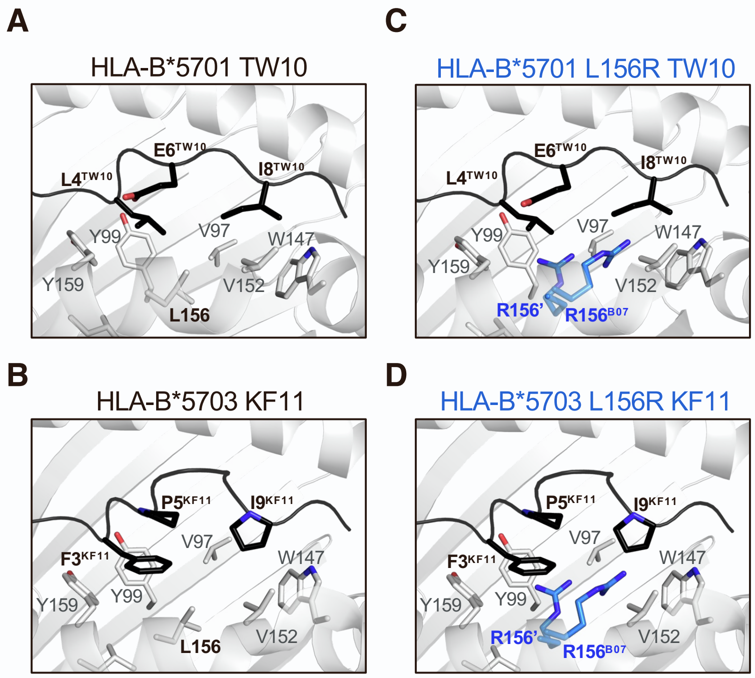


**Figure S7. Structural analysis of HLA-B*57 wildtype and L156R mutants in complex with the TW10 and KF11 peptides, related to Figure 8. (A)** Structural analysis of wild-type HLA-B*5701 presenting TW10 (PDB: 5V5M) and **(B)** the closely related wild-type HLA-B*5703 presenting KF11 (PDB: 2YPK). HLA-B*57 was shown as grey ribbon and stick, while peptides were shown in black ribbon and stick. **(C)** Structural analysis of mutant L156R HLA-B*5701-TW10 complex and **(D)** mutant L156R HLA-B*5703-KF11 complex. Two possible models of the L156R mutation were shown as blue sticks. The model of $R156^{B07}$ was constructed using the risk allele HLA-B07 (PDB: 5EO0), while the R156' model was constructed by using the Backbone Independent Rotamers function in Pymol.
